# *Limosilactobacillus balticus* sp. nov., *Limosilactobacillus agrestis* sp. nov., *Limosilactobacillus albertensis* sp. nov., *Limosilactobacillus rudii* sp. nov. and *Limosilactobacillus fastidiosus* sp. nov., five novel *Limosilactobacillus* species isolated from the vertebrate gastrointestinal tract, and proposal of six subspecies of *Limosilactobacillus reuteri* adapted to the gastrointestinal tract of specific vertebrate hosts

**DOI:** 10.1099/ijsem.0.004644

**Published:** 2021-02-03

**Authors:** Fuyong Li, Christopher C. Cheng, Jinshui Zheng, Junhong Liu, Rodrigo Margain Quevedo, Junjie Li, Stefan Roos, Michael G. Gänzle, Jens Walter

**Affiliations:** ^1^​ Department of Agricultural, Food and Nutritional Science, University of Alberta, Edmonton, Alberta, T6G 2E1, Canada; ^2^​ Department of Biological Sciences, University of Alberta, Edmonton, Alberta, T6G 2E1, Canada; ^3^​ Huazhong Agricultural University, State Key Laboratory of Agricultural Microbiology, Hubei Key Laboratory of Agricultural Bioinformatics, Wuhan, Hubei, 430070, PR China; ^4^​ Department of Molecular Sciences, Uppsala BioCenter, Swedish University of Agricultural Sciences, Uppsala, 750 07, Sweden; ^5^​ APC Microbiome Ireland, School of Microbiology, and Department of Medicine, University College Cork, Cork, T12 YT20, Ireland

**Keywords:** evolution, host adaptation, *Limosilactobacillus reuteri* subspecies, novel *Limosilactobacillus* species, phylogenetic lineages

## Abstract

Ten strains, BG-AF3-A^T^, pH52_RY, WF-MT5-A^T^, BG-MG3-A, Lr3000^T^, RRLNB_1_1, STM3_1^T^, STM2_1, WF-MO7-1^T^ and WF-MA3-C, were isolated from intestinal or faecal samples of rodents, pheasant and primate. 16S rRNA gene analysis identified them as *Limosilactobacillus reuteri *. However, average nucleotide identity and digital DNA–DNA hybridization values based on whole genomes were below 95 and 70 %, respectively, and thus below the threshold levels for bacterial species delineation. Based on genomic, chemotaxonomic and morphological analyses, we propose five novel species with the names *Limosilactobacillus balticus* sp. nov. (type strain BG-AF3-A^T^=DSM 110574^T^=LMG 31633^T^), *Limosilactobacillus agrestis* sp. nov. (type strain WF-MT5-A^T^=DSM 110569^T^=LMG 31629^T^), *Limosilactobacillus albertensis* sp. nov. (type strain Lr3000^T^=DSM 110573^T^=LMG 31632^T^), *Limosilactobacillus rudii* sp. nov. (type strain STM3_1^T^=DSM 110572^T^=LMG 31631^T^) and *Limosilactobacillus fastidiosus* sp. nov. (type strain WF-MO7-1^T^=DSM 110576^T^=LMG 31630^T^). Core genome phylogeny and experimental evidence of host adaptation of strains of *L. reuteri* further provide a strong rationale to consider a number of distinct lineages within this species as subspecies. Here we propose six subspecies of *L. reuteri *: *L. reuteri* subsp. *kinnaridis* subsp. nov. (type strain AP3^T^=DSM 110703^T^=LMG 31724^T^), *L. reuteri* subsp. *porcinus* subsp. nov. (type strain 3c6^T^=DSM 110571^T^=LMG 31635^T^), *L. reuteri* subsp. *murium* subsp. nov. (type strain lpuph1^T^=DSM 110570^T^=LMG 31634^T^), *L. reuteri* subsp. *reuteri* subsp. nov. (type strain F 275^T^=DSM 20016^T^=ATCC 23272^T^), *L. reuteri* subsp. *suis* subsp. nov. (type strain 1063^T^=ATCC 53608^T^=LMG 31752^T^) and *L. reuteri* subsp. *rodentium* subsp. nov. (type strain 100-23^T^=DSM 17509^T^=CIP 109821^T^).

## Introduction

The vertebrate gastrointestinal tract harbours diverse microbial communities that are referred to as the gut microbiota. Composed of microbes that often function as symbionts, these communities contribute substantially to the health and performance of their vertebrate hosts through the provision of nutrients and vitamins, cues for the development of the immune system and protection from bacterial and viral pathogens [1–3]. *Limosilactobacillus reuteri* (formerly *Lactobacillus reuteri *) [4], classified in the genus *Limosilactobacillus* of the family *Lactobacillaceae *, is a member of the intestinal microbiota of humans and other vertebrates and has been used as a model organism to study the adaptation of intestinal micro-organisms to their hosts [5–8]. The family *Lactobacillaceae* currently comprises more than 300 species [4], including species that are adapted to vertebrate and invertebrate hosts, free-living lactobacilli and nomadic organisms which oscillate between host-associated and environmental habitats [9]. The genus *Limosilactobacillus* (previously designated as the *L. reuteri* group) contains 17 species, including *L. alvi *, *L. antri *, *L. caviae *, *L. coleohominis *, *L. equigenerosi *, *L. fermentum *, *L. frumenti *, *L. gastricus *, *L. gorillae *, *L. ingluviei *, *L. mucosae *, *L. oris *, *L. panis *, *L. pontis *, *L. reuteri *, *L. secaliphilus* and *L. vaginalis* [4, 9, 10], and is considered adapted to vertebrates with very few exceptions (* L. fermentum* and *L. secaliphilus *) [9].

The species *L. reuteri* is extremely well studied due to its wide use as probiotics. Isolates have been obtained mainly from humans and several animal species including mice, rats, pigs, ruminants and birds, as well as fermented food [7, 8]. This species is divided into six phylogenetic clusters that show a clear association with particular vertebrate species [5–7, 11], and the genome content of these lineages is reflective of the niche characteristics in different vertebrate species [6]. These findings point to host specialization, which has been experimentally demonstrated in mice and chicken [5–7, 11]. Molecular factors that confer host specialization and allow stable persistence of *L. reuteri* in the intestinal tract of animals have been identified and relate to acid resistance, syntrophic interactions with *Bifidobacterium *, facilitation in biofilms with other lactobacilli, adhesion to non-secretory epithelia and biofilm formation, and sucrose-dependent biofilm formation [11–16]. Species in *Limosilactobacillus* including *L. reuteri* are also stable elements in food fermentation, particularly cereal fermentation [17, 18].

## Isolation and ecology

To expand our understanding of the evolution and host adaptation of *L. reuteri *, we sampled wild and zoo animals with the goal of isolating a wider array of *L. reuteri* strains. Ten strains, BG-AF3-A^T^, pH52_RY, WF-MT5-A^T^, BG-MG3-A, Lr3000^T^, RRLNB_1_1, STM3_1^T^, STM2_1, WF-MO7-1^T^ and WF-MA3-C were selected and analysed in the current study. Five of them were obtained from wild rodents during a previous study [13]: strains BG-AF3-A^T^, WF-MT5-A^T^, BG-MG3-A, WF-MO7-1^T^ and WF-MA3-C were isolated from the jejunum of yellow-necked mouse (*Apodemus flavicollis*), field vole (*Microtus agrestis*), bank vole (*Myodes glareolus*), root vole (*Microtus oeconomus*) and common vole (*Microtus arvalis*), respectively, in the Vilnius area (Lithuania) (Table 1). Strain pH52_RY was isolated from the intestine of a pheasant (*Phasianus colchicus*) in Sweden (Swedish University of Agricultural Sciences, Uppsala, Sweden), Lr3000^T^ from the stomach of a hamster in the USA by BioGaia (Stockholm, Sweden), RRLNB_1_1 from the faecal sample of a red ruffed lemur (*Varecia rubra*) raised at San Francisco Zoo (CA, USA), and STM3_1^T^ and STM2_1 from faeces of a striped mouse (*Rhabdomys pumilio*) raised at the Henry Doorly Zoo and Aquarium (Omaha, NE, USA) (Table 1). Strains were either isolated on MRS (De Man, Rogosa, Sharpe) or modified MRS (mMRS) medium, and cultivated under anaerobic conditions at 37 °C. mMRS refers to MRS supplied with 10 g l^−1^ maltose and 5 g l^−1^ fructose. Colonies were then purified, sub-cultured and preserved as glycerol stocks at −80 °C.

**Table 1.** Genomic characteristics, host origins and quality of genome assemblies of ten strains classified as novel *Limosilactobacillus* species

	* L. balticus* sp. nov.		* L. agrestis* sp. nov.		* L. albertensis* sp. nov.		* L. rudii* sp. nov.		* L. fastidiosus* sp. nov.	
	BG-AF3-A^T^	pH52_RY	WF-MT5-A^T^	BG-MG3-A	Lr3000^T^	RRLNB_1_1	STM3_1^T^	STM2_1	WF-MO7-1^T^	WF-MA3-C
Host origin	Yellow-necked mouse (*Apodemus flavicollis*)	Pheasant (*Phasianus colchicus*)	Field vole (*Microtus agrestis*)	Bank vole (*Myodes glareolus*)	Hamster	Red ruffed lemur (*Varecia rubra*)	Striped mouse (*Rhabdomys pumilio*)	Striped mouse (*Rhabdomys pumilio*)	Root vole (*Microtus oeconomus*)	Common vole (*Microtus arvalis*)
GenBank ID	GCA_014145615.1	GCA_014145605.1	GCA_014145585.1	GCA_014145545.1	GCA_014145555.1	GCA_014145525.1	GCA_014145455.1	GCA_014145435.1	GCA_014145505.1	GCA_014145425.1
IMG Genome ID	2860350408	2860354513	2860352526	2860356824	2860358791	2860361239	2860373262	2860375653	2860378031	2860379800
Genome size (bp)	2 088 966	2 169 741	1 899 103	1 863 625	2 452 361	2 354 505	2 316 339	2 307 466	1 720 134	1 728 435
Coverage (×)	874	940	937	896	768	1027	1194	889	1121	1107
G+C content (mol%)	38.3	38.2	38.0	37.9	38.8	38.5	38.5	38.5	39.1	39.1
No. of contigs	76	94	73	67	78	72	63	61	44	64
N50 (bp)	136 912	105 538	73 671	115 417	116 418	121 174	131 342	118 051	112 343	99 702
Total genes	2117	2310	1986	1966	2447	2383	2390	2377	1768	1820
No. of CDS^*^	1992	2183	1881	1845	2317	2259	2247	2233	1668	1718

^*^CDS, coding sequence.

A previous study classified several of these strains as *L. reuteri* on the basis of partial sequences of 16S rRNA genes that were >98.5 % identical to *L. reuteri* [13]. However, analysis of whole-genome sequences not only revealed that these strains fell into phylogenetic lineages that were distinct from the lineages described for *L. reuteri* [5, 7], but also showed that the average nucleotide identity (ANI) and digital DNA–DNA hybridization (dDDH) values with *L. reuteri* type strain DSM 20016^T^ ranged from 73.4 to 93.6% and from 20.8 to 54.9 %, respectively, which are below the ANI (95 %) and dDDH (70 %) thresholds that are currently accepted for a bacterial species [19–23]. We therefore concluded that these ten strains could not be assigned to any validly published species in the genus *Limosilactobacillus *. Therefore, in the present study, we propose that these strains represent five novel *Limosilactobacillus* species based on whole-genome sequencing, 16S rRNA gene sequence analysis, chemotaxonomic analysis and morphological analysis.

## Phylogeny based on the 16S rRNA gene

Genomic DNA of each of the ten strains was extracted from overnight cultures using the Wizard Genomic DNA Purification Kit (Promega) according to the manufacturer’s protocol for Gram-positive bacteria. DNA quality and quantity were estimated using a NanoDrop Spectrophotometer ND-1000 (Thermo Fisher Scientific). Libraries for whole-genome sequencing were constructed using the NEBNext Ultra II DNA Library Prep Kit for Illumina (New England Biolabs) and sequenced on the Illumina HiSeqX platform to produce paired-end reads with the length of 150 bp at the McGill University and Génome Québec Innovation Centre (Montréal, QC, Canada). Quality control (QC) of each sequencing dataset was conducted using Trimmomatic (version 0.36) [24] to trim adapters and cut low-quality bases (quality scores <20). Post-QC reads with read length no less than 100 bp were *de novo* assembled using ABySS (version 2.0) [10, 25] and contigs assigned to the PhiX sequence (NCBI accession: NC_001422.1) were removed. Only contigs with more than 200 bp were kept for downstream analysis. The completeness and the contamination of each genome assembly were estimated using CheckM [26]; the completeness and the contamination of genomes were more than 98 % and less than 2 %, respectively, demonstrating that strains or DNA from these strains were not contaminated during incubation or DNA isolation.

Genome sequences for type strains of *L. reuteri* (DSM 20016^T^; GenBank: GCA_000016825.1), *L. antri* (DSM 16041^T^; GenBank: GCA_000160835.1), *L. coleohominis* (DSM 14060^T^; GenBank: GCA_001435055.1), *L. equigenerosi* (DSM 18793^T^; GenBank: GCA_001435245.1), *L. fermentum* (DSM 20052^T^; GenBank: GCA_000159215.1), *L. frumenti* (DSM 13145^T^; GenBank: GCA_001436045.1), *L. gastricus* (DSM 16045^T^; GenBank: GCA_001434365.1), *L. gorillae* (DSM 28356^T^; GenBank: GCA_001293735.1), *L. ingluviei* (DSM 15946^T^; GenBank: GCA_001435775.1), *L. mucosae* (DSM 13345^T^; GenBank: GCA_001436025.1), *L. oris* (DSM 4864^T^; GenBank: GCA_001434465.1), *L. panis* (DSM 6035^T^; GenBank: GCA_001435935.1), *L. pontis* (DSM 8475^T^; GenBank: GCA_001435345.1), *L. secaliphilus* (DSM 17896^T^; GenBank: GCA_001437055.1) and *L. vaginalis* (DSM 5837^T^; GenBank: GCA_001435915.1) were retrieved from the GenBank database. An additional 32 genome sequences of published *L. reuteri* strains were obtained from the Joint Genome Institute (JGI) genome portal or the GenBank database (Table S1, available in the online version of this article).

**Fig. 1.**
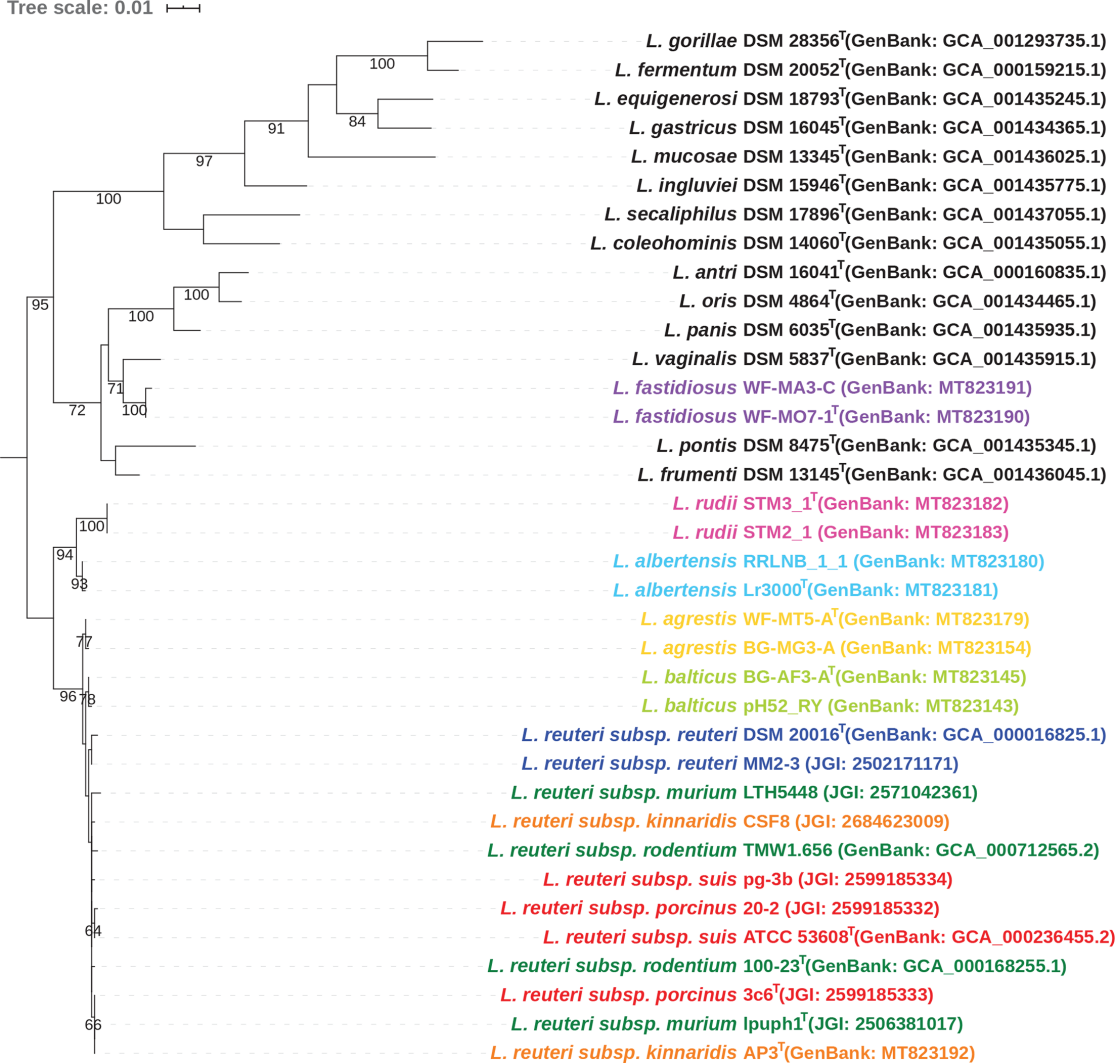
A maximum-likelihood phylogenetic tree reconstructed using 16S rRNA gene sequences. GenBank or JGI accession numbers of these genomes are provided in parentheses. The tree was inferred based on the GTR+G model with 1000 bootstrap replicates and only bootstrap values above 60 % are shown. Strains of five novel *Limosilactobacillus* species are labelled by different colours; labels of six *L. reuteri* subspecies are colour representing vertebrate host origin: green for rodents, red for pigs, blue for humans and orange for poultry. The tree was drawn with iTOL [54].

Full length (1566–1570 bp) 16S rRNA gene sequences of these strains were extracted from their draft genomes through mapping contigs to the 16S rRNA gene of *L. reuteri* DSM 20016^T^. The 16S rRNA gene similarities among these strains (Table 2) were calculated using the blast algorithm [27]. To infer the phylogeny of these ten strains and other related species in *Limosilactobacillus *, their 16S rRNA gene sequences were aligned using muscle [28] and the maximum-likelihood (ML) phylogenetic tree was inferred based on the generalized time-reversible model and the Gamma distribution (GTR+G) with 1000 bootstrap replicates through RAxML [29] (Fig. 1). Sequence similarity of strains BG-AF3-A^T^, pH52_RY, WF-MT5-A^T^, BG-MG3-A, Lr3000^T^, RRLNB_1_1, STM3_1^T^ and STM2_1 was ≥98 % when compared to *L. reuteri* DSM 20016^T^ (Table 2); these strains were closely related to *L. reuteri* according to the 16S-based phylogenetic tree (Fig. 1), which led to the initial classification of these strains to *L. reuteri* [13]. Strains WF-MO7-1^T^ and WF-MA3-C were most closely related to *L. vaginalis* (Fig. 1) and the identities of their 16S rRNA genes to *L. vaginali *s DSM 5837^T^ were 98.5 and 98.4 %, respectively (Table 2).

**Table 2.** 16S rRNA gene similarity (%) between five novel *Limosilactobacillus* species, six *L. reuteri* subspecies and other closely related species in the genus *Limosilactobacillus
*

	* L. balticus * sp. nov.		* L. agrestis * sp. nov.		* L. albertensis * sp. nov.		* L. rudii * sp. nov.		* L. fastidiosus * sp. nov.		* L. reuteri * subsp. *kinnaridis* subsp. nov.		* L. reuteri * subsp. *porcinus* subsp. nov.		* L. reuteri * subsp. *murium* subsp. nov.		* L. reuteri * subsp. *reuteri* subsp. nov.		* L. reuteri * subsp. *suis* subsp. nov.		* L. reuteri * subsp. *rodentium* subsp. nov.	
Strain	BG- AF3- A^T^	pH52_ RY	WF- MT5- A^T^	BG- MG3- A	Lr 3000^T^	RRLNB _1_1	STM3 _1^T^	STM2_1	WF-MO7 -1^T^	WF- MA3- C	AP3^T^	CSF8	3c6^T^	20–2	lpuph1^T^	LTH 5448	DSM 20016^T^	MM2 -3	ATCC 53608^T^	pg- 3b	100- 23^T^	TMW 1.656
* L. balticus * sp. nov.																						
BG-AF3-A^T^	–																					
pH52_RY	99.9	–																				
* L. agrestis * sp. nov.																						
WF-MT5-A^T^	99.8	99.8	–																			
BG-MG3-A	99.8	99.7	99.9	–																		
* L. albertensis * sp. nov.																						
Lr3000^T^	98.7	98.7	98.7	98.6	–																	
RRLNB_1_1	98.8	98.7	98.7	98.7	99.9	–																
* L. rudii * sp. nov.																						
STM3_1^T^	98.2	98.2	98.3	98.2	99.2	99.2	–															
STM2_1	98.2	98.2	98.3	98.2	99.2	99.2	100.0	–														
* L. fastidiosus * sp. nov.																						
WF-MO7-1^T^	97.1	97.1	97.2	97.1	97.3	97.3	97.3	97.3	–													
WF- MA3-C	97.0	96.9	97.1	97.0	97.2	97.1	97.2	97.2	99.9	–												
* L. reuteri * subsp. *kinnaridis* subsp. nov.																						
AP3^T^	99.8	99.7	99.7	99.6	98.6	98.7	98.2	98.2	97.3	97.1	–											
CSF8	99.8	99.7	99.7	99.6	98.5	98.5	98.2	98.2	97.2	97.1	99.9	–										
* L. reuteri* subsp. *porcinus * subsp. nov.																						
3c6^T^	99.7	99.6	99.6	99.6	98.5	98.6	98.2	98.2	97.2	97.1	99.9	99.8	–									
20–2	99.6	99.6	99.6	99.5	98.3	98.4	98.0	98.0	97.1	97.0	99.8	99.8	99.7	–								
* L. reuteri * subsp. *murium* subsp. nov.																						
lpuph1^T^	99.8	99.7	99.7	99.6	98.6	98.7	98.2	98.2	97.3	97.1	100.0	99.9	99.9	99.8	–							
LTH5448	99.6	99.6	99.6	99.6	98.3	98.4	98.0	98.0	97.0	96.9	99.8	99.8	99.7	99.6	99.8	–						
* L. reuteri * subsp. *reuteri* subsp. nov.																						
DSM 20016^T^	99.7	99.6	99.6	99.7	98.4	98.5	98.0	98.0	96.9	96.8	99.7	99.7	99.6	99.6	99.7	99.7	–					
MM2-3	99.8	99.8	99.8	99.8	98.5	98.6	98.2	98.2	97.1	96.9	99.8	99.8	99.8	99.7	99.8	99.8	99.9	–				
* L. reuteri * subsp. *suis* subsp. nov.																						
ATCC 53608^T^	99.8	99.7	99.7	99.6	98.5	98.5	98.1	98.1	97.3	97.1	99.9	99.9	99.8	99.9	99.9	99.8	99.7	99.8	–			
pg-3b	99.8	99.7	99.7	99.6	98.6	98.7	98.2	98.2	97.3	97.1	99.9	99.9	99.8	99.8	99.9	99.8	99.7	99.8	99.9	–		
* L. reuteri * subsp. *rodentium* subsp. nov.																						
100-23^T^	99.8	99.7	99.7	99.6	98.5	98.6	98.2	98.2	97.3	97.1	99.9	99.9	99.8	99.8	99.9	99.8	99.7	99.8	99.9	99.9	–	
TMW1.656	99.7	99.6	99.6	99.6	98.5	98.5	98.2	98.2	97.3	97.1	99.8	99.8	99.8	99.7	99.8	99.7	99.6	99.8	99.8	99.8	99.8	–
* L. oris * DSM 4864^T^	96.1	96.1	96.2	96.2	96.3	96.3	96.7	96.7	97.3	97.2	96.2	96.2	96.1	96.0	96.2	95.9	96.1	96.1	96.1	96.2	96.2	96.2
* L. antri * DSM 16041^T^	95.7	95.7	95.7	95.7	96.0	95.9	96.3	96.3	97.2	97.1	95.8	95.7	95.7	95.6	95.8	95.5	95.7	95.7	95.7	95.8	95.8	95.8
* L. panis * DSM 6035^T^	96.1	96.0	96.2	96.1	96.2	96.2	96.4	96.4	97.5	97.3	96.1	96.1	96.1	95.9	96.1	95.9	96.0	96.1	96.0	96.1	96.1	96.0
* L. pontis * DSM 8475^T^	97.3	97.2	97.3	97.2	97.3	97.2	96.9	96.9	97.6	97.5	97.3	97.2	97.2	97.0	97.3	97.0	97.1	97.2	97.1	97.3	97.3	97.1
* L. vaginalis * DSM 5837^T^	96.7	96.6	96.8	96.7	96.9	96.9	96.8	96.8	98.5	98.4	96.7	96.6	96.6	96.4	96.7	96.4	96.6	96.6	96.6	96.7	96.7	96.7
* L. frumenti * DSM 13145^T^	97.3	97.2	97.2	97.1	97.4	97.4	97.1	97.1	98.4	98.3	97.1	97.1	97.1	96.9	97.1	96.9	97.0	97.1	97.0	97.1	97.1	97.0
* L. coleohominis * DSM 14060^T^	95.0	94.9	95.2	95.2	94.9	94.9	95.2	95.2	95.6	95.5	95.0	95.0	95.0	94.9	95.0	94.9	95.0	95.1	95.0	95.0	95.0	94.9

## Genomic analyses

**Fig. 2.**
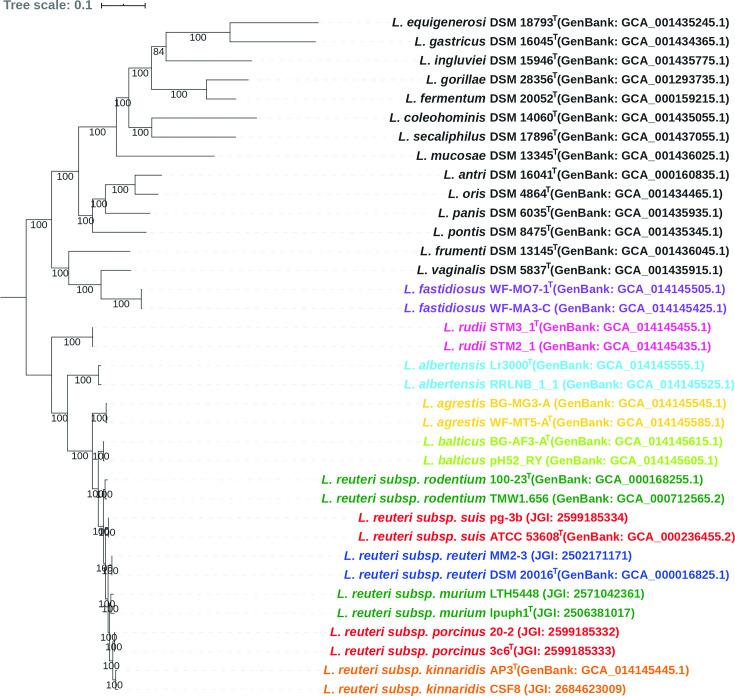
A maximum-likelihood phylogenetic tree reconstructed using core genes (*n*=100) identified from whole-genome sequences, showing the evolutionary relationships among five novel *Limosilactobacillus* species, six *L. reuteri* subspecies and other recognized species in the genus *Limosilactobacillus *. GenBank or JGI accession numbers of these genomes are provided in parentheses. The tree was inferred based on the GTR+G model with 1000 bootstrap replicates and only bootstrap values above 60 % are shown. Strains of five novel *Limosilactobacillus* species are labelled by different colours; labels of six *L. reuteri* subspecies are colour representing vertebrate host origin: green for rodents, red for pigs, blue for humans and orange for poultry. The tree was drawn with iTOL [54].

Draft genomes of the ten *Limosilactobacillus* strains were annotated using the Integrated Microbial Genomes (IMG) system from the Joint Genome Institute [30] and genomic characteristics of these strains are summarized in Table 1. The G+C content of the draft genomes ranged from 37.9 to 39.1 mol% and the genome size ranged from 1.72 to 2.45 Mbp (Table 1). A phylogenetic tree of these strains was reconstructed based on the alignments of the genes shared in all genomes (*n*=100). Briefly, draft genomes of the ten *Limosilactobacillus* strains, published *L. reuteri* strains and other type strains of the genus *Limosilactobacillus* were re-annotated using Prokka with default settings [31]. The Roary pipeline was applied to identify core genes based on these re-annotated assemblies [32] and concatenated core gene alignments were used as the input for RAxML to reconstruct the maximum-likelihood (ML) tree using the GTR+G model with 1000 bootstrap replicates [29]. The core-gene-based phylogenetic tree confirmed that strains BG-AF3-A^T^, pH52_RY, WF-MT5-A^T^, BG-MG3-A, Lr3000^T^, RRLNB_1_1, STM3_1^T^ and STM2_1 were closely related to *L. reuteri* lineages but formed distinct phylogenetic clades, while the strains WF-MO7-1^T^ and WF-MA3-C clustered with *L. vaginalis* (Fig. 2).

To determine which of the distinct phylogenetic clades should be considered as separate species, pairwise ANI values were calculated based on entire genomes using JSpeciesWS and JSpecies with the blast algorithm [21, 33] and pairwise dDDH values were estimated using the Genome-To-Genome Distance Calculator (GGDC) [22]. Pairwise ANI and dDDH values of the ten strains of *Limosilactobacillus* with other species of *Limosilactobacillus* and two representative strains from each of six *L. reuteri* lineages are shown in Tables 3 and 4, respectively. The ANI and dDDH values of strains from different phylogenetic clades to type strains of known *Limosilactobacillus* species were lower than 93.6 and 54.9 %, respectively, and therefore lower than the threshold for assignment to established species (95 % of ANI and 70 % of dDDH) [19–23]. In contrast, strains within the same phylogenetic clade had ANI values between 97.0 and 100 %, and dDDH values between 77.0 and 100 % (Tables 3 and 4). Strains WF-MO7-1^T^ and WF-MA3-C were most closely related to the type strain of *L. vaginalis* (DSM 5837^T^) (Fig. 2), but their ANI and dDDH values with *L. vaginalis* DSM 5837^T^ were below 82 and 26 %, respectively (Tables 3 and 4). Strains Lr3000^T^ and RRLNB_1_1, as well as STM3_1^T^ and STM2_1 were most closely related to *L. reuteri *, but the ANI and dDDH values of these strains with *L. reuteri* DSM 20016^T^ were below 82 and 25 %, respectively (Tables 3 and 4). The ANI and dDDH values of strains BG-AF3-A^T^ and pH52_RY as well as WF-MT5-A^T^ and BG-MG3-A with *L. reuteri* DSM20016^T^ were much higher (90.7–93.6 % of ANI and 43.2–54.9 % of dDDH) but still below 94 and 55 %, respectively. These analyses demonstrated that the ten strains represent five novel species.

**Table 3.** Average nucleotide identity values (%) between five novel *Limosilactobacillus* species, six *L. reuteri* subspecies and other closely related species in the genus *Limosilactobacillus
*

	* L. balticus* sp. nov.		* L. agrestis* sp. nov.		* L. albertensis* sp. nov.		* L. rudii* sp. nov.		* L. fastidiosus* sp. nov.		* L. reuteri * subsp. *kinnaridis* subsp. nov.		* L. reuteri * subsp. *porcinus* subsp. nov.		* L. reuteri * subsp. *murium* subsp. nov.		* L. reuteri * subsp. *reuteri* subsp. nov.		* L. reuteri * subsp. *suis* subsp. nov.		* L. reuteri * subsp. *rodentium* subsp. nov.	
Strain	BG-AF3-A^T^	pH52_RY	WF-MT5-A^T^	BG-MG3-A	Lr3000^T^	RRLNB_1_1	STM3_1^T^	STM2_1	WF-MO7- 1^T^	WF-MA3-C	AP3^T^	CSF8	3c6^T^	20–2	lpuph1^T^	LTH5448	DSM 20016^T^	MM2-3	ATCC 53608^T^	pg-3b	100-23^T^	TMW1.656
* L. balticus * sp. nov.																						
BG-AF3-A^T^	–																					
pH52_RY	98.3	–																				
* L. agrestis * sp. nov.																						
WF-MT5-A^T^	91.0	91.2	–																			
BG-MG3-A	91.1	91.4	98.8	–																		
* L. albertensis * sp. nov.																						
Lr3000^T^	81.9	82.1	81.3	81.8	–																	
RRLNB_1_1	81.6	81.9	81.4	81.7	97.0	–																
* L. rudii * sp. nov.																						
STM3_1^T^	79.5	79.2	79.1	79.2	79.8	79.7	–															
STM2_1	79.5	79.2	79.1	79.2	79.7	79.7	100.0	–														
* L. fastidiosus * sp. nov.																						
WF-MO7-1^T^	73.4	73.4	73.0	73.2	73.0	73.0	73.4	73.4	–													
WF-MA3-C	73.5	73.5	73.2	73.2	73.3	73.2	73.6	73.5	99.7	–												
* L. reuteri * subsp. *kinnaridis* subsp. nov.																						
AP3^T^	93.2	93.3	90.6	90.7	82.2	82.4	79.4	79.4	73.5	73.5	–											
CSF8	93.2	93.2	90.5	90.7	82.1	81.9	79.2	79.2	73.3	73.3	98.2	–										
* L. reuteri * subsp. *porcinus* subsp. nov.																						
3c6^T^	92.8	93.0	90.4	90.6	81.5	81.5	79.0	79.0	73.2	73.2	96.3	96.6	–									
20–2	92.7	93.0	90.3	90.6	81.8	81.6	79.0	79.0	73.0	73.4	96.3	96.6	99.1	–								
* L. reuteri * subsp. *murium* subsp. nov.																						
lpuph1^T^	93.0	93.1	90.5	90.7	81.5	81.5	79.1	79.1	73.3	73.3	95.9	95.8	96.4	96.3	–							
LTH5448	93.0	93.1	90.5	90.7	81.5	81.5	79.0	79.0	73.3	73.2	95.5	95.7	95.9	95.8	97.0	–						
* L. reuteri * subsp. *reuteri* subsp. nov.																						
DSM 20016^T^	93.6	93.6	90.7	90.8	81.7	81.9	79.4	79.4	73.5	73.4	95.4	95.8	96.1	96.0	96.5	96.5	–					
MM2-3	93.5	93.5	90.7	90.7	81.7	81.7	79.2	79.2	73.4	73.4	95.3	95.8	96.0	96.0	96.4	96.3	100.0	–				
* L. reuteri* subsp. *suis* subsp. nov.																						
ATCC53608^T^	94.1	94.2	90.9	91.0	82.2	82.4	79.4	79.4	73.6	73.6	95.4	95.2	94.7	94.7	95.6	95.6	95.7	95.7	–			
pg-3b	93.9	94.0	90.6	90.8	81.5	81.7	79.2	79.2	73.1	73.2	95.2	95.3	95.1	95.1	95.5	95.7	96.1	96.1	99.0	–		
* L. reuteri * subsp. *rodentium* subsp. nov.																						
100-23^T^	93.8	94.0	90.8	90.9	81.7	81.8	79.1	79.2	73.3	73.5	94.8	94.7	94.8	94.8	95.6	95.8	96.2	96.1	96.3	96.2	–	
TMW1.656	94.2	94.3	90.6	90.7	81.4	81.5	78.8	78.9	72.8	73.0	94.6	94.7	94.9	94.9	95.5	95.6	95.9	95.9	96.2	95.9	97.3	–
* L. oris * DSM 4864^T^	71.6	71.1	70.9	71.1	71.4	71.0	71.5	71.4	71.3	71.5	71.6	71.3	71.0	70.9	71.1	71.0	71.5	71.0	72.1	70.9	71.5	70.8
* L. antri * DSM 16041^T^	70.7	70.6	70.5	70.8	71.1	70.6	71.0	70.8	71.0	71.1	71.3	70.8	70.7	70.7	70.7	70.7	71.1	70.6	71.5	70.5	71.0	70.3
* L. panis * DSM 6035^T^	71.7	71.7	71.5	71.7	72.0	71.6	71.8	71.7	71.8	72.1	72.3	71.9	71.5	71.4	71.7	71.5	72.0	71.7	72.2	71.5	72.2	71.4
* L. pontis * DSM 8475^T^	70.8	70.5	70.4	70.7	70.8	70.6	70.6	70.7	71.0	71.1	71.4	70.8	70.5	70.9	70.7	70.6	71.1	70.7	71.3	70.6	71.0	70.4
* L. vaginalis * DSM 5837^T^	73.4	73.3	73.1	73.2	73.0	73.3	73.5	73.4	81.5	81.7	74.4	73.5	73.1	73.1	73.2	73.4	73.6	73.0	73.6	73.0	73.9	72.9
* L. frumenti * DSM 13145^T^	72.4	72.4	72.4	72.6	72.0	72.4	72.5	72.5	74.0	74.0	73.0	72.6	72.4	72.3	72.5	72.5	73.1	72.3	73.1	72.1	73.2	71.9
* L. coleohominis * DSM 14060^T^	71.1	71.1	70.6	71.1	71.2	71.1	71.6	71.4	71.2	71.1	72.0	71.0	70.9	70.8	71.0	70.7	71.3	70.8	71.6	70.9	71.6	70.3

**Table 4.** Digital DNA–DNA hybridization values (%) between five novel *Limosilactobacillus* species, six *L. reuteri* subspecies and other closely related species in the genus *Limosilactobacillus
*

Strain	* L. balticus* sp. nov.		* L. agrestis* sp. nov.		* L. albertensis* sp. nov.		* L. rudii* sp. nov.		* L. fastidiosus* sp. nov.		* L. reuteri * subsp. *kinnaridis* subsp. nov.		* L. reuteri * subsp. *porcinus* subsp. nov.		* L. reuteri * subsp. *murium* subsp. nov.		* L. reuteri * subsp. *reuteri* subsp. nov.		* L. reuteri * subsp. *suis* subsp. nov.		* L. reuteri * subsp. *rodentium* subsp. nov.	
	BG-AF3-A^T^	pH52_RY	WF-MT5-A^T^	BG-MG3-A	Lr3000^T^	RRLNB_1_1	STM3_1^T^	STM2_1	WF-MO7-1^T^	WF-MA3-C	AP3^T^	CSF8	3c6^T^	20–2	lpuph1^T^	LTH5448	DSM 20016^T^	MM2-3	ATCC 53608^T^	pg-3b	100-23^T^	TMW1.656
* L. balticus * sp. nov.																						
BG-AF3-A^T^	–																					
pH52_RY	89.0	–																				
* L. agrestis * sp. nov.																						
WF-MT5-A^T^	45.0	45.2	–																			
BG-MG3-A	45.1	45.2	91.2	–																		
* L. albertensis * sp. nov.																						
Lr3000^T^	25.4	25.5	24.5	25.2	–																	
RRLNB_1_1	25.1	25.1	24.6	25.0	77.0	–																
* L. rudii * sp. nov.																						
STM3_1^T^	22.5	22.1	22.3	22.3	22.8	22.6	–															
STM2_1	22.5	22.1	22.3	22.3	22.8	22.6	100.0	–														
* L. fastidiosus * sp. nov.																						
WF-MO7-1^T^	20.5	20.4	20.6	20.8	20.5	20.2	20.8	20.8	–													
WF-MA3-C	20.5	20.3	20.8	20.9	20.8	20.5	20.9	20.9	98.6	–												
* L. reuteri * subsp. *kinnaridis* subsp. nov.																						
AP3^T^	52.8	53.0	42.4	42.4	25.5	25.5	22.4	22.4	20.5	20.5	–											
CSF8	53.6	53.1	42.5	42.8	25.3	25.0	22.4	22.4	20.4	20.4	89.7	–										
* L. reuteri* subsp. *porcinus* subsp. nov.																						
3c6^T^	52.2	52.3	42.9	43.0	24.7	24.7	22.3	22.3	20.6	20.6	72.0	75.5	–									
20–2	51.7	51.9	42.6	43.1	25.1	24.7	22.3	22.3	20.4	21.1	71.3	74.8	95.0	–								
* L. reuteri * subsp. *murium* subsp. nov.																						
lpuph1^T^	52.5	52.5	43.2	43.1	24.7	24.6	22.5	22.5	20.4	20.5	68.2	68.1	71.2	70.7	–							
LTH5448	52.7	52.8	42.8	42.7	24.6	24.6	22.3	22.3	20.3	20.1	67.0	67.2	69.5	69.0	75.6	–						
* L. reuteri * subsp. *reuteri* subsp. nov.																						
DSM 20016^T^	54.9	54.7	43.2	43.2	24.9	24.9	22.5	22.5	20.8	20.8	65.1	68.4	70.0	70.1	71.5	72.0	–					
MM2-3	54.6	54.4	43.0	42.9	24.6	24.7	22.1	22.1	20.3	20.3	64.6	68.2	69.5	69.8	70.9	71.4	99.9	–				
* L. reuteri * subsp. *suis* subsp. nov.																						
ATCC53608^T^	57.4	57.8	43.7	43.8	25.8	25.9	22.6	22.6	21.2	21.2	65.1	64.2	62.4	62.1	65.5	65.7	68.2	68.1	–			
pg-3b	57.0	57.5	43.5	43.6	24.9	24.7	22.4	22.4	20.1	20.1	63.8	64.0	64.3	64.1	65.3	67.0	70.4	70.7	94.9	–		
* L. reuteri * subsp. *rodentium* subsp. nov.																						
100-23^T^	57.1	57.7	43.6	43.7	25.3	25.1	22.7	22.7	21.0	21.0	62.0	61.5	63.0	62.8	65.6	66.3	69.0	68.6	70.7	70.9	–	
TMW1.656	58.6	58.7	42.9	42.9	24.6	24.5	22.0	22.0	19.4	19.4	61.3	61.1	63.3	63.2	65.6	66.0	67.8	67.9	70.7	69.8	80.1	–
* L. oris * DSM 4864^T^	22.2	20.6	20.0	20.3	20.0	20.5	20.3	20.3	21.8	21.8	20.8	20.1	19.9	19.8	20.2	19.9	21.3	20.2	22.0	20.1	21.7	19.7
* L. antri * DSM 16041^T^	20.2	19.8	18.9	19.1	21.1	20.5	19.7	19.7	21.9	21.8	21.1	20.1	19.7	19.9	20.4	20.3	20.8	19.8	21.1	19.9	21.3	19.7
* L. panis * DSM 6035^T^	19.2	19.1	19.4	19.5	19.8	19.5	20.0	20.0	21.8	21.8	19.5	19.2	19.1	18.9	19.1	19	19.7	18.8	20.1	19.3	20.2	18.9
* L. pontis* DSM 8475^T^	18.7	18.6	18.5	18.6	18.7	18.6	19.5	19.5	20.5	20.7	19.6	18.9	18.9	19.1	18.4	18.4	19.6	18.8	19.9	18.8	19.4	18.2
* L. vaginalis * DSM 5837^T^	20.6	20.5	20.9	20.9	20.9	20.7	20.7	20.7	25.0	25.3	21.6	20.8	21.0	20.8	20.7	21.1	21.7	20.6	21.2	20.1	21.9	20.6
* L. frumenti * DSM 13145^T^	20.8	20.9	20.1	20.2	20.1	19.9	20.6	20.6	20.0	20.2	20.8	20.5	19.9	19.8	20.4	20.6	21.1	19.8	21.8	20.6	23.0	19.7
* L. coleohominis * DSM 14060^T^	26.2	25.7	24.2	23.3	26.0	25.0	27.1	27.2	24.9	25.5	23.3	23.6	23.9	23.9	23.8	24.4	28.0	24.4	27.5	23.4	26.7	22.2

**Fig. 3.**
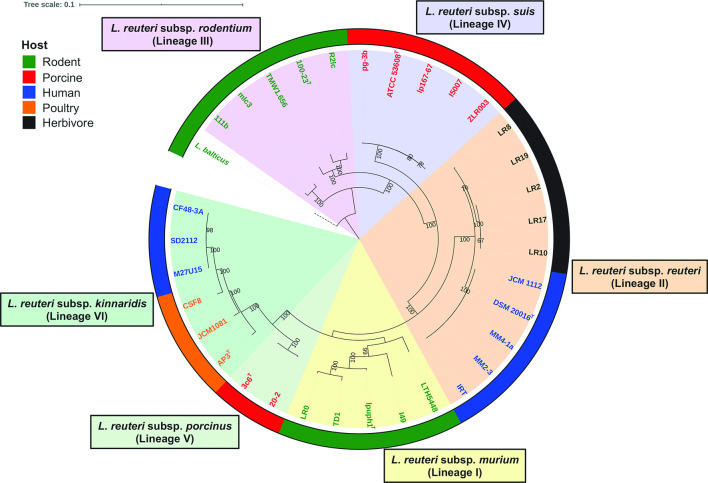
A maximum-likelihood phylogenetic tree reconstructed using core genes (*n*=100) identified from whole-genome sequences, showing the evolutionary relationships among six *L. reuteri* subspecies. The tree was reconstructed using 33 *L *. *reuteri* genomes available in public databases (*n*=6 for *L. reuteri* subsp. *kinnaridis *, *n*=2 for *L. reuteri* subsp. *porcinus *, *n*=5 for *L. reuteri* subsp. *murium *, *n*=10 for *L. reuteri* subsp. *reuteri *, *n*=5 for *L. reuteri* subsp. *suis* and *n*=5 for *L. reuteri* subsp. *rodentium *) and *L. balticus* BG-AF3-A^T^ was used as an outgroup. Further information on the involved genome sequences is listed in Table S1. The tree was inferred based on the GTR+G model with 1000 bootstrap replicates and only bootstrap values above 60 % are shown. The tree was drawn with iTOL [54].

**Fig. 4.**
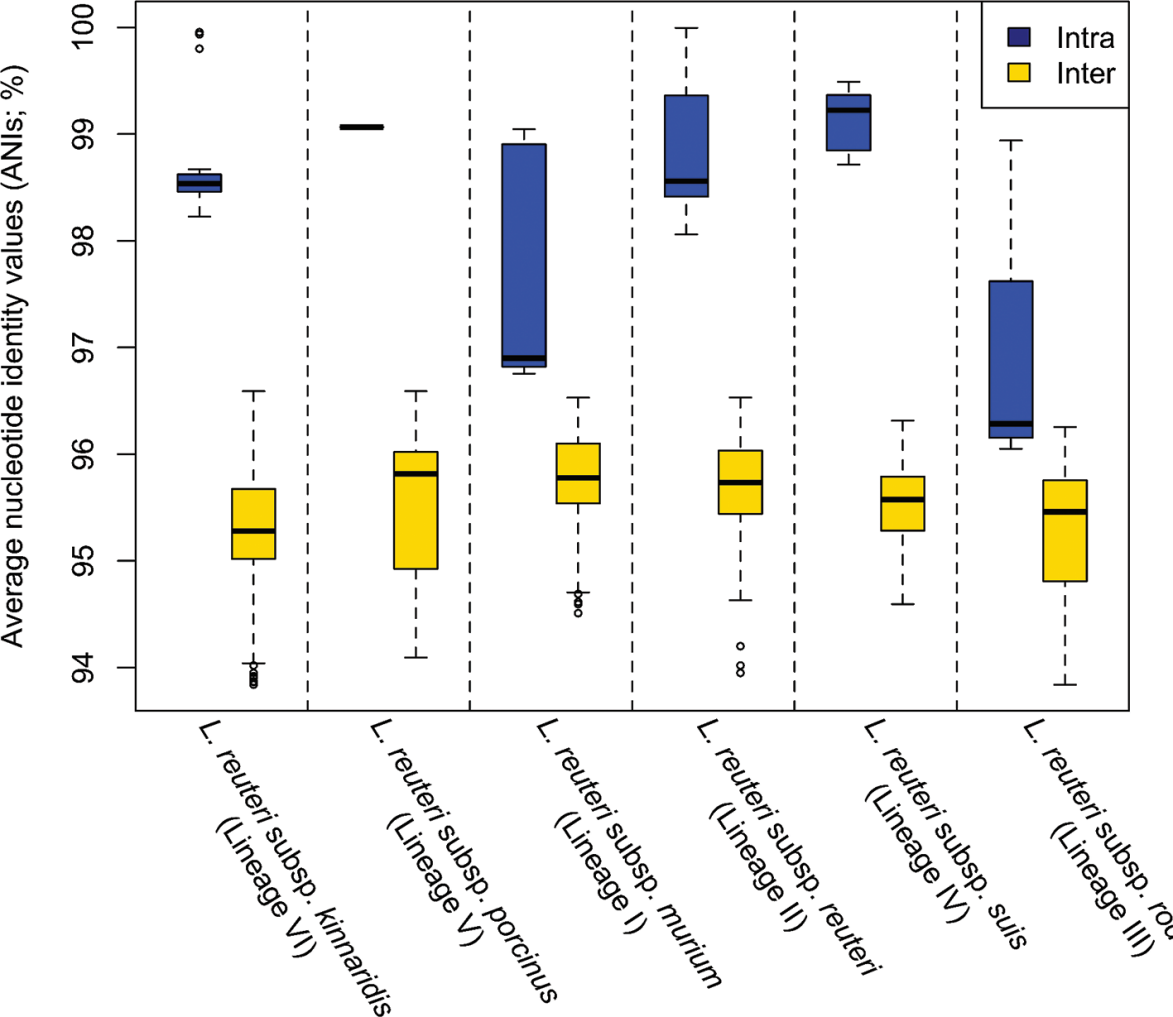
Pairwise average nucleotide identity values (ANI; %) of genome sequences belonging to the same or different *L. reuteri* subspecies. ANI values within the same subspecies and between different subspecies were calculated for 33 *L. reuteri* genomes available in public databases (*n*=6 for *L. reuteri* subsp. *kinnaridis *, *n*=2 for *L. reuteri* subsp. *porcinus *, *n*=5 for *L. reuteri* subsp. *murium *, *n*=10 for *L. reuteri* subsp. *reuteri *, *n*=5 for *L. reuteri* subsp. *suis* and *n*=5 for *L. reuteri* subsp. *rodentium *). Further information on the involved genome sequences is listed in Table S1.

Representative strains of *L. reuteri* that represent the six host-adapted phylogenetic lineages were not sufficiently resolved by the analysis based on 16S rRNA gene sequences (Fig. 1) but formed distinct phylogenetic clades using the core-gene-based analysis (Fig. 2), which is consistent with previous findings [5, 7]. However, ANI and dDDH values of strains belonging to different *L. reuteri* lineages were from 94.6 to 96.6 % and from 61.1 to 75.5 %, respectively, indicating genetic dissimilarity (Tables 3 and 4). To gain further insight into the genetic dissimilarities and evolutionary relationships among strains belonging to different *L. reuteri* lineages, a core-gene-based phylogenetic tree was reconstructed for 33 *L *. *reuteri* genomes available in public databases (Table S1) with the BG-AF3-A^T^ strain as an outlier. In accordance with previous analyses that were based on multi-locus sequence analysis (MLSA), amplified-fragment length polymorphism (AFLP) or core genome phylogeny [5, 7, 34], strains of *L. reuteri* clustered into six cohesive host-adapted lineages (Fig. 3). Pairwise ANI values of *L. reuteri* strains within the same lineages (98.5±1.0 %; mean±SD) were higher than those of strains belonging to different lineages (95.5±0.6 %), with little overlap (Fig. 4). These analyses provide phylogenetic and genomic support for the assignation of subspecies status to the six *L. reuteri* lineages.

## Physiology and chemotaxonomy

Carbohydrate utilization of the five novel *Limosilactobacillus* species and the six *L. reuteri* subspecies was determined with the API 50 CH system (bioMérieux) following the manufacturer’s instructions; results are shown in Table 5. Aspects of the fermentation phenotypes of the strains are in agreement with presence/absence patterns of the phosphofructokinase gene, the mannitol dehydrogenase gene (*mdh*) and the gene (*adhE*) encoding a two-domain enzyme combining acetyl coenzyme A (acetyl-CoA) and alcohol dehydrogenase domains in the annotated draft genomes [10]. The phosphofructokinase gene was absent, while the *mdh* and *adhE* genes were present in all of these ten strains, which matches the gene content of heterofermentative lactobacilli but differs from the gene content of homofermentative lactobacilli [4]. This *in silico* prediction was verified for all five proposed type strains (BG-AF3-A^T^, WF-MT5-A^T^, Lr3000^T^, STM3_1^T^ and WF-MO7-1^T^) by observation of gas formation from glucose in MRS broth with Durham tubes. The lactate isomers fermented from glucose of these five putative type strains were determined using QuantiQuik d-Lactic Acid Quick Test Strips and QuantiQuik l-Lactic Acid Quick Test Strips (BioAssay Systems): both d-lactate and l-lactate were produced by these five strains from the glucose fermentation. To evaluate the optimal temperature conditions for growth, the five putative type strains were incubated anaerobically in MRS broth (in the 96-well microplate; each well was overlaid with paraffin to keep the oxygen out) at 15, 30, 37 and 45 °C, and OD_600_ was measured hourly using SpectraMax M3 Multi-Mode Microplate Readers (Molecular Devices) for 24 h (Fig. S1). None of these five strains grew at 15 °C. Optimum growth of BG-AF3-A^T^ and Lr3000^T^ occurred at 45 °C, while 37 °C was the optimum temperature for the growth of WF-MT5-A^T^ and STM3_1^T^ (Fig. S1). WF-MO7-1^T^ did not grow in the 96-well microplate (Fig. S1), but its growth occurred at 30, 37 and 45 °C (optimum) in Falcon 15 ml tubes (data not shown). To investigate the pH range for growth, the pH of MRS broth was adjusted to 4.0, 4.5, 5.0, 5.5, 6.0, 6.5, 7.0, 7.5 and 8.0. Growth of BG-AF3-A^T^, WF-MT5-A^T^, Lr3000^T^ and STM3_1^T^ occurred at pH 4.0–8.0, and growth of WF-MO7-1^T^ occurred at pH 4.5–7.5. The Gram-staining pattern of each strain was checked using Bactident Aminopeptidase strips (Merck) and the potassium hydroxide test (KOH test) [35]. The ten *Limosilactobacillus* strains were aminopeptidase- and KOH-negative, suggesting that they are Gram-positive bacteria. The catalase activity was examined using the standard methods as described previously [36] and further confirmed through checking the absence of catalase and NADPH peroxidase genes in the annotated draft genomes [37], which showed that these ten *Limosilactobacillus* strains are catalase-negative.

**Table 5.** Carbohydrate utilization phenotypes of five novel *Limosilactobacillus* species, six *L. reuteri* subspecies and *L. vaginalis
* Carbohydrates with negative or not determined results for all strains: glycerol, erythritol, d-arabinose, l-xylose, d-adonitol, methyl β-d-xylopyranoside, l-sorbose, l-rhamnose, dulcitol, inositol, d-mannitol, d-sorbitol, methyl α-d-mannopyranoside, *N*-acetylglucosamine, amygdalin, arbutin, salicin, cellobiose, trehalose, inulin, melezitose, starch, glycogen, xylitol, gentiobiose, d-lyxose, d-tagatose, d-fucose, l-fucose, d-arabitol, l-arabitol, potassium 2-ketogluconate and potassium 5-ketogluconate. Grey shading, acid production from sugar indicated; white, no acid production from sugar indicated or not determined.

Characteristic	*L. Balticus* sp. nov.		* L. agrestis * sp. nov.		*L. Albertensis* sp. nov.		*L. Rudii* sp. nov.		*L.* *Fastidiosus* sp. nov.		*L. Reuteri* subsp. *kinnaridis* subsp. nov.		*L. Reuteri* subsp. *porcinus* subsp. nov.		*L. Reuteri* subsp. *murium* subsp. nov.*		*L. Reuteri* subsp. *reuteri* subsp. nov.^†^		*L. Reuteri* subsp.*suis* subsp. nov.		*L. Reuteri* subsp. *rodentium* subsp. nov.*		* L. vaginalis* ^†^
	BG-AF3-A^T^	pH52 _RY	WF-MT5-A^T^	BG-MG3-A	Lr 3000^T^	RRLNB _1_1	STM3_1^T^	STM2_1	WF-MO7-1^T^	WF-MA3-C	AP3^T^	CSF8	3 c6^T^	20–2	lpuph1^T^	LTH 5448	DSM 20016^T^	MM2-3	ATCC 53608^T^	lp167-67	100–23^T^	TMW 1.656	DSM 5837^T^
l-Arabinose																							
d-Ribose																							
d-Xylose																							
d-Galactose																							
d-Glucose																							
d-Fructose																							
d-Mannose																							
Methyl- α-d- glucopyranoside																							
Aesculin/ ferric citrate																							
Maltose																							
Lactose																							
Melibiose																							
Sucrose																							
Raffinose																							
Turanose																							
Potassium gluconate																							

*API 50 CH test data of *L. reuteri* subsp. *murium* (lpuph1^T^ and LTH5448) and *L. reuteri* subsp. *rodentium* (100-23^T^ and TMW1.656) were obtained from [51].

†API 50 CH test data of *L. reuteri* DSM 20016^T^ and *L. vaginalis* DSM 5837^T^ were retrieved from the Bacterial Diversity Metadatabase (Bac*Dive*; https://bacdive.dsmz.de/).

Peptidoglycan structure of five putative type strains (BG-AF3-A^T^, WF-MT5-A^T^, Lr3000^T^, STM3_1^T^ and WF-MO7-1^T^) was analysed as described previously [38]. The cell morphology of cells grown on MRS agar plates for 24–48 h under anaerobic conditions was observed with a Scope-A1 microscope (Carl Zeiss) (Fig. S2). Results are presented in the species description section. Cellular fatty acid profiles were generated using the Sherlock MIS (midi) system by the Identification Service of the DSMZ, for which TSBA40 was used for the initial analysis and TSBA6 was used for calculation. Fatty acid profiles of these ten strains showed variations: C_16 : 0_ was the major fatty acid in BG-AF3-A^T^, pH52_RY, WF-MT5-A^T^, BG-MG3-A, STM3_1^T^, STM2_1, WF-MO7-1^T^ and WF-MA3-C, while summed feature 7 (combination of C_19 : 1_ *ω*6*c* and/or C_19 : 0_ cyclo *ω*10*c*) was more abundant than other fatty acids in Lr3000^T^ and RRLNB_1_1 (from the same putative novel species) (Table 6). Cluster analysis based on fatty acid compositions suggested that two strains representing each putative novel species had generally more similar fatty acid profiles to each other, compared to strains from other putative novel species, with the only exceptions of BG-AF3-A^T^ and pH52_RY (Table 6, Fig. S3). This further confirms the delineation of five novel species.

**Table 6.** Cellular fatty acid profiles of the ten strains classified as novel *Limosilactobacillus* species

Fatty acid (%)	* L. balticus* sp. nov		* L. agrestis* sp. nov.		* L. albertensis* sp. nov.		* L. rudii* sp. nov.		* L. fastidiosus* sp. nov.		* L. reuteri *	* L. vaginalis *
	BG-AF3-A^T^	pH52_RY	WF-MT5-A^T^	BG-MG3-A	Lr3000^T^	RRLNB_1_1	STM3_1^T^	STM2_1	WF-MO7-1^T^	WF-MA3-C	DSM 20016^T^	DSM 5837^T^
C_14 : 0_	2.6	3.6	1.8	5.3	0.5	0.6	1.8	2.0	7.6	7.2	2.3	7.4
iso-C_15 : 0_	0.0	0.0	0.0	0.0	0.0	0.0	0.0	0.0	0.0	0.2	0.0	0.0
iso-C_16 : 0_	0.0	0.0	0.0	0.0	0.0	0.0	0.0	0.0	0.0	0.7	0.0	0.0
C_16 : 0_	42.3	32.4	48.3	50.4	16.5	16.9	42.2	46.0	33.4	30.2	37.4	23.7
C_17 : 0_ cyclo	0.0	0.0	0.0	0.0	0.0	0.0	0.0	0.0	0.5	0.0	0.0	0.0
C_16 : 0_ 3-OH	0.0	0.4	0.0	0.0	0.0	0.0	0.0	0.0	0.5	0.6	0.0	0.0
C_18 : 1_ *ω*9c	6.4	21.7	5.7	7.3	31.4	30.1	6.5	6.6	17.2	21.2	6.7	33.3
C_18 : 0_	3.0	2.5	5.0	2.5	3.3	3.3	5.3	5.7	1.7	2.2	7.1	0.0
C_18 : 1_ *ω*7c 11-methyl	0.0	0.0	0.4	0.0	0.0	0.0	0.0	0.0	0.0	0.0	0.0	0.0
C_17 : 0_ 3-OH	0.0	0.0	0.0	0.0	0.0	0.0	0.0	0.3	0.0	0.0	0.0	0.0
iso-C_19 : 0_	1.0	1.3	0.4	0.0	2.3	2.5	1.3	1.2	0.0	1.0	0.0	0.0
C_19 : 0_ cyclo *ω*8*c*	18.9	8.4	20.4	13.6	0.0	0.0	14.2	15.7	0.0	0.0	7.0	0.0
Summed feature 3†	1.5	1.7	1.5	1.9	1.2	1.3	1.3	1.5	2.7	2.0	3.0	4.4
Summed feature 5†	0.0	0.0	0.0	0.0	0.0	0.0	0.0	0.0	0.0	0.0	17.1	0.0
Summed feature 7†	14.5	19.3	13.8	15.1	36.8	38.4	20.6	21.2	31.4	30.1	5.4	15.7
Summed feature 8†	9.9	8.7	2.6	4.0	8.1	7.0	7.0	0.0	5.2	4.6	13.9	15.5

*Cellular fatty acid data of *L. reuteri* DSM 20016^T^ and *L. vaginalis* DSM 5837^T^ were retrieved from the Bacterial Diversity Metadatabase (Bac*Dive*; https://bacdive.dsmz.de/).

†Summed feature 3 comprises C_16 : 1_ *ω*7*c* and/or C_16 : 1_ *ω*6c; summed feature 5 comprises C_18 : 2_ *ω*6,9*c* and/or C_18 : 0_ anteiso; summed feature 7 comprises C_19 : 1_ *ω*6*c* and/or C_19 : 0_ cyclo *ω*10*c*; summed feature 8 is an unknown combination of C_18 : 1_ *ω*7*c* and/or C_18 : 1_ *ω*6*c*.

## Proposal of novel species and subspecies within *L. reuteri*

According to aforementioned polyphasic analyses and the chemotaxonomic and phenotypic characterization, we propose that these ten strains represent five novel species of the genus *Limosilactobacillus *. We propose the names *Limosilactobacillus balticus* sp. nov. (type strain BG-AF3-A^T^=DSM 110574^T^=LMG 31633^T^), *Limosilactobacillus agrestis* sp. nov. (type strain WF-MT5-A^T^=DSM 110569^T^=LMG 31629^T^), *Limosilactobacillus albertensis* sp. nov. (type strain Lr3000^T^=DSM 110573^T^=LMG 31632^T^), *Limosilactobacillus rudii* sp. nov. (type strain STM3_1^T^=DSM 110572^T^=LMG 31631^T^) and *Limosilactobacillus fastidiosus* sp. nov. (type strain WF-MO7-1^T^=DSM 110576^T^=LMG 31630^T^) for these novel species.

Based on the cohesive phylogenetic lineages that showed little overlap in ANI values and the experimental proof of differences in host adaptation between the phylogenetic lineages [5, 7, 11] and characteristic physiological differences related to the utilization of glycerol [6, 8, 16, 39, 40], decarboxylation of histidine [34], synthesis of folate [34] and the expression of mucus-binding large surface proteins [8, 41, 42], we also propose that the six phylogenetic lineages within *L. reuteri* represent six subspecies. The following names are proposed: *L. reuteri* subsp. *kinnaridis* subsp. nov. (type strain AP3^T^=DSM 110703^T^=LMG 31724^T^), *L. reuteri* subsp. *porcinus* subsp. nov. (type strain 3c6^T^=DSM 110571^T^=LMG 31635^T^), *L. reuteri* subsp. *murium* subsp. nov. (type strain lpuph1^T^=DSM 110570^T^=LMG 31634^T^), *L. reuteri* subsp. *reuteri* subsp. nov. (type strain DSM 20016^T^=ATCC 23272^T^=F 275^T^ [original designation]), *L. reuteri* subsp. *suis* subsp. nov. (type strain ATCC 53608^T^=LMG 31752^T^=1063^T^ [original designation]) and *L. reuteri* subsp. *rodentium* subsp. nov. (type strain 100-23^T^=DSM 17509^T^=CIP 109821^T^).

## Description of *Limosilactobacillus balticus* sp. nov.

*Limosilactobacillus balticus* (bal.ti′cus. L. adj. *balticus* pertaining to the Baltic region where the type strain was isolated).

Cells are Gram-positive, non-motile, non-spore-forming, catalase-negative and heterofermentative. Cells are rod-shaped, measuring 0.9–3.0×0.6–1.0 µm. Colonies of the type strain BG-AF3-A^T^ on MRS agar plate incubated at the anaerobic condition at 37 °C for 2 days are whitish, opaque, raised, circular and entire, with a diameter of 1.2–3.2 mm; no colony appears on MRS agar plate incubated at the aerobic condition at 37 °C for 2 days. d-Lactate, l-lactate and gas are produced from glucose fermentation by the type strain BG-AF3-A^T^. Growth occurs at 30, 37 and 45 °C (optimum), but not at 15 °C. Growth occurs at pH 4.0–8.0 in MRS broth. For BG-AF3-A^T^, the most abundant fatty acid is C_16 : 0_, followed by C_19 : 0_ cyclo *ω*8*c*. Acid is produced from l-arabinose, d-ribose, d-xylose, d-galactose, d-glucose, aesculin, maltose, lactose, melibiose, sucrose and raffinose; acid is not produced from d-fructose, d-mannose, methyl α-d-glucopyranoside, potassium gluconate, glycerol, erythritol, d-arabinose, l-xylose, d-adonitol, methyl β-d-xylopyranoside, l-sorbose, l-rhamnose, dulcitol, inositol, d-mannitol, d-sorbitol, methyl α-d-mannopyranoside, *N*-acetylglucosamine, amygdalin, arbutin, salicin, cellobiose, trehalose, inulin, melezitose, starch, glycogen, xylitol, gentiobiose, turanose, d-lyxose, d-tagatose, d-fucose, l-fucose, d-arabitol, l-arabitol, potassium 2-ketogluconate or potassium 5-ketogluconate. The cell-wall peptidoglycan of BG-AF3-A^T^ contains the amino acids alanine (Ala), glutamic acid (Glu), lysine (Lys), and aspartic acid (Asp), with the molar ratio 1.3 (Ala):0.8 (Asp):1.0 (Glu):1.0 (Lys), suggesting the cell-wall peptidoglycan type A4α l-Lys–d-Asp by [38]. The DNA G+C content of BG-AF3-A^T^ is 38.3 mol%. The type strain is BG-AF3-A^T^ (=DSM 110574^T^=LMG 31633^T^), which was isolated from the jejunum of yellow-necked mouse (*Apodemus flavicollis*) caught in the Vilnius area in Lithuania [13].

## Description of *Limosilactobacillus agrestis* sp. nov.

*Limosilactobacillus agrestis* (a.gres′tis. L. masc. adj. *agrestis*, wild, referring to the isolation of the type strain from wild rodents).

Cells are Gram-positive, non-motile, non-spore-forming, catalase-negative and heterofermentative. Cells are rod-shaped, measuring 0.9–2.7×0.6–0.8 µm. Colonies of the type strain WF-MT5-A^T^ on MRS agar plate incubated at the anaerobic condition at 37 °C for 2 days are yellowish, translucent, flat, circular shape but irregular edge, with a diameter of 2.2–5.2 mm; no colony appears on MRS agar plate incubated at the aerobic condition at 37 °C for 2 days. d-Lactate, l-lactate and gas are produced from glucose fermentation by the type strain WF-MT5-A^T^. Cell growth occurs at 37 °C, slow growth is observed at 30 and 45 °C and no growth occurs at 15 °C. Growth occurs at pH 4.0–8.0 in MRS broth. For WF-MT5-A^T^, the major fatty acids are C_16 : 0_, followed by C_19 : 0_ cyclo *ω*8*c*. Acid is produced from l-arabinose and aesculin; the fermentation of d-ribose, d-galactose, d-glucose, maltose, melibiose, sucrose and raffinose is strain-specific; acid is not produced from d-xylose, d-fructose, d-mannose, methyl α-d-glucopyranoside, lactose, potassium gluconate, glycerol, erythritol, d-arabinose, l-xylose, d-adonitol, methyl β-d-xylopyranoside, l-sorbose, l-rhamnose, dulcitol, inositol, d-mannitol, d-sorbitol, methyl α-d-mannopyranoside, *N*-acetylglucosamine, amygdalin, arbutin, salicin, cellobiose, trehalose, inulin, melezitose, starch, glycogen, xylitol, gentiobiose, turanose, d-lyxose, d-tagatose, d-fucose, l-fucose, d-arabitol, l-arabitol, potassium 2-ketogluconate or potassium 5-ketogluconate. The cell-wall peptidoglycan of WF-MT5-A^T^ contains the amino acids alanine (Ala), glutamic acid (Glu), lysine (Lys), aspartic acid (Asp), serine (Ser) and threonine (Thr), with the molar ratio 1.1 (Ala):0.8 (Asp):1.0 (Glu):0.7 (Lys):0.1 (Thr):0.1 (Ser), suggesting the cell-wall peptidoglycan type A4α l-Lys–d-Asp by [38]. The DNA G+C content of WF-MT5-A^T^ is 38.0 mol%. The type strain is WF-MT5-A^T^ (=DSM 110569^T^=LMG 31629^T^), which was isolated from the jejunum of field vole (*Microtus agrestis*) caught in the Vilnius area in Lithuania [13].

## Description of *Limosilactobacillus albertensis* sp. nov.

*Limosilactobacillus albertensis* (al.ber.ten′sis. N.L. masc. adj. *albertensis*, pertaining to Alberta, a province of Canada where the isolates were characterized and identified).

Cells are Gram-positive, non-motile, non-spore forming, catalase-negative and heterofermentative. Cells are rod-shaped, measuring 0.8–2.4×0.6–1.2 µm. Colonies of the type strain Lr3000^T^ on MRS agar plate incubated at the anaerobic condition at 37 °C for 2 days are whitish, opaque, raised, circular and entire, with a diameter of 0.8–1.5 mm; colonies on MRS agar plate at the aerobic condition show similar morphological characteristics as colonies on MRS agar incubated anaerobically, but with a smaller diameter of 0.5–1 mm. d-Lactate, l-lactate and gas are produced from glucose fermentation by the type strain Lr3000^T^. Cell growth occurs at 30, 37 and 45 °C (optimum), but not at 15 °C. Growth occurs at pH 4.0–8.0 in MRS broth. The major fatty acids of Lr3000^T^ are summed feature 7 (combination of C_19 : 1_ *ω*6*c* and/or C_19 : 0_ cyclo *ω*10*c*) and C_18 : 1_ *ω*9c, followed by C_16 : 0_. Acid is produced from l-arabinose, d-ribose, d-xylose, d-galactose, d-glucose, methyl α-d-glucopyranoside, aesculin, maltose, lactose, melibiose, sucrose and raffinose; the fermentation of potassium gluconate is strain-specific; acid is not produced from d-fructose, d-mannose, glycerol, erythritol, d-arabinose, l-xylose, d-adonitol, methyl β-d-xylopyranoside, l-sorbose, l-rhamnose, dulcitol, inositol, d-mannitol, d-sorbitol, methyl α-d-mannopyranoside, *N*-acetylglucosamine, amygdalin, arbutin, salicin, cellobiose, trehalose, inulin, melezitose, starch, glycogen, xylitol, gentiobiose, turanose, d-lyxose, d-tagatose, d-fucose, l-fucose, d-arabitol, l-arabitol, potassium 2-ketogluconate or potassium 5-ketogluconate. The cell-wall peptidoglycan of Lr3000^T^ contains the amino acids alanine (Ala), glutamic acid (Glu), lysine (Lys) and aspartic acid (Asp), with the molar ratio 1.1 (Ala):0.8 (Asp):1.0 (Glu):1.0 (Lys), suggesting the cell-wall peptidoglycan type A4α l-Lys–d-Asp [38]. The DNA G+C content of Lr3000^T^ is 38.8 mol%. The type strain, Lr3000^T^ (=DSM 110573^T^=LMG 31632^T^), was isolated from the stomach of a hamster in the USA.

## Description of *Limosilactobacillus rudii* sp. nov.

*Limosilactobacillus rudii* [ru′di.i.i. N.L. gen. n. *rudii* of Rudi (Vogel), in recognition of the German scientist Rudi F. Vogel, in recognition of his significant contributions to the taxonomy of lactic acid bacteria as well as the technology and microbial ecology of fermented foods].

Cells are Gram-positive, non-motile, non-spore-forming, catalase-negative and heterofermentative. Cells are rod-shaped, measuring 1.1–2.7×0.7–1.2 µm. Colonies of the type strain STM3_1^T^ on MRS agar plate incubated under the anaerobic condition at 37 °C for 2 days are whitish, opaque, raised, circular and entire, with a diameter of 1.0–2.2 mm; colonies on MRS agar plate at the aerobic condition show similar morphological characteristics as colonies on MRS agar incubated anaerobically, but with a smaller diameter of 0.4–0.8 mm. d-Lactate, l-lactate and gas are produced from glucose fermentation by the type strain STM3_1^T^. Cell growth occurs at 30 and 37 °C (optimum), but not at 15 or 45 °C. Growth occurs at pH 4.0–8.0 in MRS broth. The major fatty acid of STM3_1^T^ is C_16 : 0_, followed by summed feature 7 (combination of C_19 : 1_ *ω*6*c* and/or C_19 : 0_ cyclo *ω*10*c*) and C_19 : 0_ cyclo *ω*8*c*. Acid is produced from l-arabinose, d-ribose, d-xylose, d-galactose, d-glucose, aesculin, maltose, lactose, melibiose, sucrose and raffinose; acid is not produced from d-fructose, d-mannose, methyl α-d-glucopyranoside, potassium gluconate, glycerol, erythritol, d-arabinose, l-xylose, d-adonitol, methyl β-d-xylopyranoside, l-sorbose, l-rhamnose, dulcitol, inositol, d-mannitol, d-sorbitol, methyl α-d-mannopyranoside, *N*-acetylglucosamine, amygdalin, arbutin, salicin, cellobiose, trehalose, inulin, melezitose, starch, glycogen, xylitol, gentiobiose, turanose, d-lyxose, d-tagatose, d-fucose, l-fucose, d-arabitol, l-arabitol, potassium 2-ketogluconate or potassium 5-ketogluconate. The cell-wall peptidoglycan of STM3_1^T^ contains the amino acids alanine (Ala), glutamic acid (Glu), lysine (Lys), aspartic acid (Asp), serine (Ser) and threonine (Thr), with the molar ratio 1.1 (Ala):1.0 (Asp):1.0 (Glu):0.8 (Lys):0.1 (Thr), suggesting the cell-wall peptidoglycan type A4α l-Lys–d-Asp by [38]. The DNA G+C content of STM3_1^T^ is 38.5 mol%. The type strain, STM3_1^T^ (=DSM 110572^T^=LMG 31631^T^), was isolated from the faecal sample of striped mouse (*Rhabdomys pumilio*) raised at Henry Doorly Zoo and Aquarium (Omaha, NE, USA).

## Description of *Limosilactobacillus fastidiosus* sp. nov.

*Limosilactobacillus fastidiosus* (fas.ti.di.o′sus. L. masc. adj. *fastidious,* fastidious, referring to the fastidious growth requirements of the type strain).

Cells are Gram-positive, non-motile, non-spore-forming, catalase-negative and heterofermentative. Cells are rod-shaped, measuring 0.9–3.0×0.6–0.9 µm. Colonies of the type strain WF-MO7-1^T^ on MRS agar plate incubated at the anaerobic condition at 37 °C for 2 days are whitish, opaque, raised, circular and entire, with a diameter of 1.2–2.2 mm; no colony appears on MRS agar plate incubated at the aerobic condition at 37 °C for 2 days. d-Lactate, l-lactate and gas are produced from glucose fermentation by the type strain WF-MO7-1^T^. No growth occurs in 96-well microplate at 15, 30, 37 or 45 °C; in Falcon 15 ml tubes, cell growth occurs at 30, 37 and 45 °C (optimum) but not at 15 °C. Growth occurs at pH 4.5–7.5 in MRS broth. The most abundant fatty acids of WF-MO7-1^T^ are C_16 : 0_, summed feature 7 (combination of C_19 : 1_ *ω*6*c* and/or C_19 : 0_ cyclo *ω*10*c*) and C_18 : 1_ *ω*9*c*. Acid is produced from l-arabinose and aesculin; acid production from d-galactose, d-glucose, d-fructose, maltose, lactose, melibiose and raffinose is strain-specific; acid is not produced from d-ribose, d-xylose, d-mannose, methyl α-d-glucopyranoside, sucrose, potassium gluconate, glycerol, erythritol, d-arabinose, l-xylose, d-adonitol, methyl β-d-xylopyranoside, l-sorbose, l-rhamnose, dulcitol, inositol, d-mannitol, d-sorbitol, methyl α-d-mannopyranoside, *N*-acetylglucosamine, amygdalin, arbutin, salicin, cellobiose, trehalose, inulin, melezitose, starch, glycogen, xylitol, gentiobiose, turanose, d-lyxose, d-tagatose, d-fucose, l-fucose, d-arabitol, l-arabitol, potassium 2-ketogluconate or potassium 5-ketogluconate. The cell-wall peptidoglycan of WF-MO7-1^T^ contains the amino acids alanine (Ala), glutamic acid (Glu), aspartic acid (Asp) and ornithine (Orn), with the molar ratio 1.5 (Ala):0.9 (Asp):1.0 (Glu):1.1 (Orn), suggesting the cell-wall peptidoglycan type A4α l-Orn–d-Asp by [38]. The DNA G+C content of WF-MO7-1^T^ is 39.1 mol%. The type strain is WF-MO7-1^T^ (=DSM 110576^T^=LMG 31630^T^), which was isolated from the jejunum of root vole (*Microtus oeconomus*) caught in the Vilnius area in Lithuania [13].

## Description of *Limosilactobacillus reuteri* subsp. *reuteri* subsp. nov.

*Limosilactobacillus reuteri* subsp. *reuteri* (reu′te.ri. N.L. gen. n. *reuteri*, of Reuter; named for G. Reuter, a German bacteriologist after whom the species *L. reuteri* was named).

*L. reuteri* strains clustered in lineage II (Fig. 3) belong to *L. reuteri* subsp. *reuteri* and they were isolated from humans and herbivores [7, 43]. Strains of this subspecies have ANI values of 98.1–100.0 % with each other and ANI values of 94.0–96.5 % with other *L. reuteri* strains belonging to different subspecies (Fig. 4). Acid is produced from l-arabinose, d-ribose, d-galactose, d-glucose, maltose, lactose, melibiose, sucrose and raffinose; acid production from potassium gluconate is strain-specific; acid is not produced from d-xylose, d-fructose, d-mannose, methyl α-d-glucopyranoside, aesculin, glycerol, erythritol, d-arabinose, l-xylose, d-adonitol, methyl β-d-xylopyranoside, l-sorbose, l-rhamnose, dulcitol, inositol, d-mannitol, d-sorbitol, methyl α-d-mannopyranoside, *N*-acetylglucosamine, amygdalin, arbutin, salicin, cellobiose, trehalose, inulin, melezitose, starch, glycogen, xylitol, gentiobiose, turanose, d-lyxose, d-tagatose, d-fucose, l-fucose, d-arabitol, l-arabitol, potassium 2-ketogluconate or potassium 5-ketogluconate. Phylogenetic analyses based on the core genes identified in this study (Fig. 3) and previous studies [5, 43], AFLP and MLSA (using concatenated sequences of *ddl*, *pkt*, *leuS*, *gyrB*, *dltA*, *rpoA* and *recA* genes) [7], suggest that these strains are genetically homogeneous [8]. Strains of this subspecies possess the *pdu-cbi-cob-hem* cluster (*pdu* cluster) [6, 8], which equips them with the ability to utilize 1,2-propanediol and glycerol as electron acceptors [16, 39, 40] and to produce the antimicrobial compound reuterin [8]. They also produce histamine from histidine that has been linked to their anti-inflammatory phenotype [34]. Strains belonging to this subspecies have been considered as immunosuppressive because they could suppress the proinflammatory cytokines tumour necrosis factor (TNF), monocyte chemoattractant protein (MCP)-1, interleukin (IL)-1β and IL-12, as well as suppress intestinal inflammation [34]. The type strain, DSM 20016^T^ (=ATCC 23272^T^=F 275^T^ [original designation]), was isolated from the gastrointestinal tract of an adult human [6, 44, 45], with a DNA G+C content of 38.9 mol%.

## Description of *Limosilactobacillus reuteri* subsp. *kinnaridis* subsp. nov.

*Limosilactobacillus reuteri* subsp. *kinnaridis* (kin.na′ri.dis. N.L. gen.n. *kinnaridis* of Kinnaris, referring to kinnaris, half-bird, half-woman creatures of South-East Asian mythology and reflecting occurrence of strains of this subspecies in birds and in humans. The name also reflects the use of this subspecies in probiotics, as according to south-east Asian mythology, Kinnaris are believed to come from the Himalayas and watch over the well-being of humans in times of trouble or danger).

*L. reuteri* strains clustered in lineage VI (Fig. 3) belong to *L. reuteri* subsp. *kinnaridis* and they were isolated from poultry and humans [5, 7]. Strains of this subspecies have ANI values of 98.2–100.0 % with each other and ANI values of 93.8–96.6 % with other *L. reuteri* strains belonging to different subspecies (Fig. 4). Acid is produced from d-ribose, d-galactose, d-glucose, maltose, lactose, melibiose, sucrose, raffinose and potassium gluconate; acid production from l-arabinose, methyl α-d-glucopyranoside and turanose is strain-specific; acid is not produced from d-xylose, d-fructose, d-mannose, aesculin, glycerol, erythritol, d-arabinose, l-xylose, d-adonitol, methyl β-d-xylopyranoside, l-sorbose, l-rhamnose, dulcitol, inositol, d-mannitol, d-sorbitol, methyl α-d-mannopyranoside, *N*-acetylglucosamine, amygdalin, arbutin, salicin, cellobiose, trehalose, inulin, melezitose, starch, glycogen, xylitol, gentiobiose, d-lyxose, d-tagatose, d-fucose, l-fucose, d-arabitol, l-arabitol, potassium 2-ketogluconate or potassium 5-ketogluconate. Phylogenetic analyses based on the core genes identified in this study (Fig. 3) and a previous study [5], AFLP and MLSA (using concatenated sequences of *ddl*, *pkt*, *leuS*, *gyrB*, *dltA*, *rpoA* and *recA* genes) [7] indicate that strains clustered in this lineage are adapted to poultry and also occur in humans. Experimental test has revealed that strains of *L. reuteri* subsp. *kinnaridis* displayed elevated fitness in chickens but not in humans [5], suggesting that this subspecies is autochthonous of chicken and share an evolutionary history with poultry. Strains of this subspecies possess the *pdu-cbi-cob-hem* cluster (*pdu* cluster) [6, 8], which equips them with the ability to utilize 1,2-propanediol and glycerol as electron acceptors [16, 39, 40] and to produce the broad-spectrum antimicrobial compound reuterin [8, 34]. These strains are immunostimulatory; specifically, they stimulate the production of IL-7, IL-12 and IL-13, but suppress the production of IL-5 [34]. In addition, strains belonging to this subspecies synthesize folate *de novo* [34]. The type strain, AP3^T^ (=DSM 110703^T^=LMG 31724^T^), was isolated from the gastrointestinal tract of an Argus Pheasant, with a DNA G+C content of 38.6 mol%.

## Description of *Limosilactobacillus reuteri* subsp. *porcinus* subsp. nov.

*Limosilactobacillus reuteri* subsp. *porcinus* (por.ci′nus. L. masc. adj. *porcinus* of swine, referring to the host origin of most strains of this subspecies being swine).

*L. reuteri* strains clustered in lineage V (Fig. 3) belong to *L. reuteri* subsp. *porcinus* and they were isolated from pigs [5, 7]. Strains (3c6^T^ and 20-2) of this subspecies have an ANI value of 99.1 % with each other and ANI values of 93.8–96.6 % with other *L. reuteri* strains belonging to different subspecies (Fig. 4). Acid is produced from d-ribose, d-galactose, d-glucose, maltose, lactose, melibiose, sucrose, raffinose and potassium gluconate; acid production from methyl-α-d-glucopyranoside is strain-specific; acid is not produced from l-arabinose, d-xylose, d-fructose, d-mannose, aesculin, glycerol, erythritol, d-arabinose, l-xylose, d-adonitol, methyl β-d-xylopyranoside, l-sorbose, l-rhamnose, dulcitol, inositol, d-mannitol, d-sorbitol, methyl α-d-mannopyranoside, *N*-acetylglucosamine, amygdalin, arbutin, salicin, cellobiose, trehalose, inulin, melezitose, starch, glycogen, xylitol, gentiobiose, turanose, d-lyxose, d-tagatose, d-fucose, l-fucose, d-arabitol, l-arabitol, potassium 2-ketogluconate or potassium 5-ketogluconate. Phylogenetic analyses based on the core genes identified in this study (Fig. 3) and previous studies [5, 43, 46], AFLP and MLSA (using concatenated sequences of *ddl*, *pkt*, *leuS*, *gyrB*, *dltA*, *rpoA* and *recA* genes) [7] indicate that strains clustered in this lineage are pig-specific. Both 3c6^T^ and 20-2 possess the *pdu-cbi-cob-hem* cluster (*pdu* cluster) [5, 46], which equips them with the ability to utilize 1,2-propanediol and glycerol as electron acceptors [16, 39, 40] and to produce the antimicrobial compound reuterin [8]. The type strain, 3c6^T^ (=DSM 110571^T^=LMG 31635^T^), was isolated from porcine gastrointestinal tract [7, 46], with a DNA G+C content of 38.6 mol%.

## Description of *Limosilactobacillus reuteri* subsp. *murium* subsp. nov.

*Limosilactobacillus reuteri* subsp. *murium* (mu′ri.um. L. plur. gen. n. *murium* of mice, referring to the adaptation of strains of the subspecies to rodents including mice).

*L. reuteri* strains clustered in lineage I (Fig. 3) belong to *L. reuteri* subsp. *murium* and they were isolated from rodents [5–7]. Strains of this subspecies have ANI values of 96.8–99.1 % with each other and ANI values of 94.5–96.5 % with other *L. reuteri* strains belonging to different subspecies (Fig. 4). Acid is produced from l-arabinose, d-ribose, d-galactose, d-glucose, maltose, lactose, melibiose, sucrose and raffinose; acid production from potassium gluconate is strain-specific; acid is not produced from d-xylose, d-fructose, d-mannose, methyl α-d-glucopyranoside, aesculin, glycerol, erythritol, d-arabinose, l-xylose, d-adonitol, methyl β-d-xylopyranoside, l-sorbose, l-rhamnose, dulcitol, inositol, d-mannitol, d-sorbitol, methyl α-d-mannopyranoside, N-acetylglucosamine, amygdalin, arbutin, salicin, cellobiose, trehalose, inulin, melezitose, starch, glycogen, xylitol, gentiobiose, turanose, d-lyxose, d-tagatose, d-fucose, l-fucose, d-arabitol, l-arabitol, potassium 2-ketogluconate or potassium 5-ketogluconate. Phylogenetic analyses based on the core genes identified in this study (Fig. 3) and a previous studies [5], AFLP and MLSA (using concatenated sequences of *ddl*, *pkt*, *leuS*, *gyrB*, *dltA*, *rpoA* and *recA* genes) [7] indicate that strains clustered in this lineage are rodent-specific. Strains of *L. reuteri* subsp. *murium* displayed elevated fitness in mice through the colonization and biofilm formation on the forestomach epithelium [5, 7, 11], suggesting that their evolution with rodents was adaptive and led to host specificity. Large surface proteins (>750 aa) exist among strains belonging to this subspecies, which involve in epithelial adhesion and biofilm formation [6]. Strains of this subspecies produce the enzyme urease for acid resistance and rarely produce the antimicrobial compound reuterin [6, 8]. The type strain, lpuph1^T^ (=DSM 110570^T^=LMG 31634^T^), was isolated from mouse gastrointestinal tract [6, 7], with a DNA G+C content of 38.4 mol%.

## Description of *Limosilactobacillus reuteri subsp. suis* subsp. nov.

*Limosilactobacillus reuteri* subsp. *suis* (su′is. L. gen. n. *suis*, of swine, reflecting the host origin of most strains of this subspecies being the swine intestinal tract).

*L. reuteri* strains clustered in lineage IV (Fig. 3) belong to *L. reuteri* subsp. *porcinus* and were isolated from pig [5, 7]. Strains belonging to this subspecies have ANI values of 98.7–99.5 % with each other and ANI values of 94.6–96.3 % with other *L. reuteri* strains belonging to different subspecies (Fig. 4). Acid is produced from l-arabinose, d-ribose, d-xylose, d-galactose, d-glucose, maltose, lactose, melibiose, sucrose and raffinose; acid is not produced from d-fructose, d-mannose, methyl α-d-glucopyranoside, aesculin, potassium gluconate, glycerol, erythritol, d-arabinose, l-xylose, d-adonitol, methyl β-d-xylopyranoside, l-sorbose, l-rhamnose, dulcitol, inositol, d-mannitol, d-sorbitol, methyl α-d-mannopyranoside, *N*-acetylglucosamine, amygdalin, arbutin, salicin, cellobiose, trehalose, inulin, melezitose, starch, glycogen, xylitol, gentiobiose, turanose, d-lyxose, d-tagatose, d-fucose, l-fucose, d-arabitol, l-arabitol, potassium 2-ketogluconate or potassium 5-ketogluconate. Phylogenetic analyses based on the core genes identified in this study (Fig. 3) and previous studies [5, 43, 46, 47], AFLP and MLSA (using concatenated sequences of *ddl*, *pkt*, *leuS*, *gyrB*, *dltA*, *rpoA* and *recA* genes) [7] indicate that strains clustered in this lineage are pig-specific. A mucus-binding protein (Mub) that could bind mucus and/or IgA [8, 41, 42] exists within this subspecies and it specifically supports the colonization of this subspecies to the porcine gastrointestinal tract. Strains within this subspecies have been applied as probiotics to improve porcine intestinal health, enhance production, prevent diarrhoea, release stress and immune modulation [48]. The type strain, ATCC 53608^T^ (=LMG 31752^T^=1063^T^ [original designation]), was isolated from porcine gastrointestinal tract [7, 49, 50], with a DNA G+C content of 39.0 mol%.

## Description of *Limosilactobacillus reuteri* subsp. *rodentium* subsp. nov.

*Limosilactobacillus reuteri* subsp. *rodentium* (ro.den′ti.um. L. pl. gen. n. *rodentium* of gnawing animals, reflecting adaptation of the subspecies to rodents).

*L. reuteri* strains clustered in lineage III (Fig. 3) belong to *L. reuteri* subsp. *rodentium* and were mainly isolated from rodents [5–7]. Strains of this subspecies have ANI values of 96.1–98.9 % with each other and ANI values of 93.8–96.3 % with other *L. reuteri* strains belonging to different subspecies (Fig. 4). Acid is produced from d-ribose, d-galactose, d-glucose, maltose, lactose, melibiose, sucrose, raffinose and potassium gluconate; acid production from l-arabinose and d-xylose is strain-specific; acid is not produced from d-fructose, d-mannose, methyl α-d-glucopyranoside, aesculin, glycerol, erythritol, d-arabinose, l-xylose, d-adonitol, methyl β-d-xylopyranoside, l-sorbose, l-rhamnose, dulcitol, inositol, d-mannitol, d-sorbitol, methyl α-d-mannopyranoside, *N*-acetylglucosamine, amygdalin, arbutin, salicin, cellobiose, trehalose, inulin, melezitose, starch, glycogen, xylitol, gentiobiose, turanose, d-lyxose, d-tagatose, d-fucose, l-fucose, d-arabitol, l-arabitol, potassium 2-ketogluconate or potassium 5-ketogluconate. Phylogenetic analyses based on the core genes identified in this study (Fig. 3) and a previous study [5], AFLP and MLSA (using concatenated sequences of *ddl*, *pkt*, *leuS*, *gyrB*, *dltA*, *rpoA* and *recA* genes) [7] indicate that strains clustered in this lineage including the sourdough isolates are rodent-specific. Strains of *L. reuteri* subsp. *rodentium* displayed elevated fitness in mice through the colonization and biofilm formation on the forestomach epithelium [5, 7, 11], suggesting adaptive evolution with rodents that led to host specificity. Large surface proteins (>750 aa) exist among strains belonging to this subspecies, which involve in epithelial adhesion and biofilm formation [6]. A xylose operon is highly conserved for this subspecies, especially for strains originating from rodents [6], and thus most strains of this subspecies could metabolize xylose that is an important substrate for gut bacteria [51]. In addition, strains of this subspecies produce the enzyme urease for acid resistance and rarely produce the antimicrobial compound reuterin [6, 8]. Sourdough isolates of this subspecies (LTH2584, TMW1.106, TMW1.112 and TMW1.656) produce reutericyclin, a unique antimicrobial tetramic acid with activity against Gram-positive bacteria [52]. The type strain, 100-23^T^ (=DSM 17509^T^=CIP 109821^T^), was isolated from the rat gastrointestinal tract [11, 53], with a DNA G+C content of 38.7 mol%.

## Supplementary Data

Supplementary material 1
