# Supplementary material for: Limosilactobacillus balticus sp. nov., Limosilactobacillus agrestis sp. nov., Limosilactobacillus albertensis sp. nov., Limosilactobacillus rudii sp. nov. and Limosilactobacillus fastidiosus sp. nov., five novel Limosilactobacillus species isolated from the vertebrate gastrointestinal tract, and proposal of six subspecies of Limosilactobacillus reuteri adapted to the gastrointestinal tract of specific vertebrate hosts
Source: Int J Syst Evol Microbiol. 2021 Feb 3;71(2):004644. doi: 10.1099/ijsem.0.004644 (PMC8346765; doi:10.1099/ijsem.0.004644)
Supplement: Supplementary material 1 [file ijsem-71-644-s001.pdf]

Supplementary Information

Description of *Limosilactobacillus balticus* sp. nov., *Limosilactobacillus agrestis* sp. nov., *Limosilactobacillus albertensis* sp. nov., *Limosilactobacillus rudii* sp. nov., *Limosilactobacillus fastidiosus* sp. nov., five novel *Limosilactobacillus* species isolated from the vertebrate gastrointestinal tract, and proposal of six subspecies of *Limosilactobacillus reuteri* adapted to the gastrointestinal tract of specific vertebrate hosts

Fuyong Li, Christopher C. Cheng, Jinshui Zheng, Junhong Liu, Rodrigo Margain Quevedo, Junjie Li, Stefan Roos, Michael G. Gänzle, Jens Walter

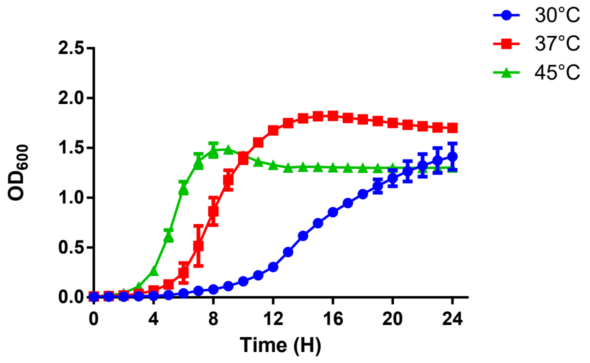

a. *Limosilactobacillus balticus* BG-AF3-A^T

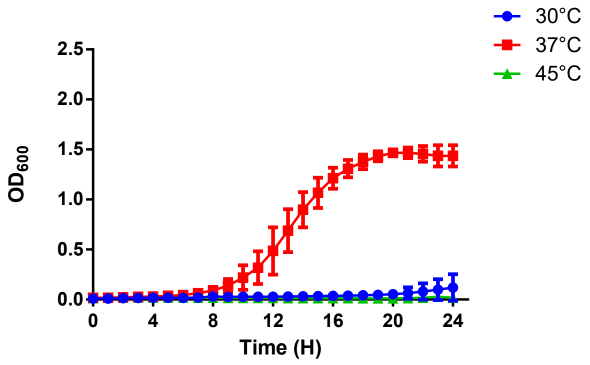

b. *Limosilactobacillus agrestis* WF-MT5-A^T

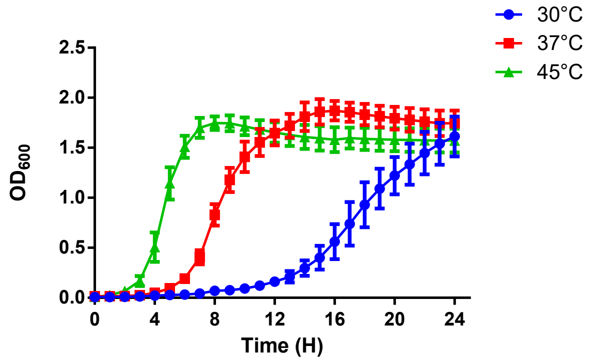

c. *Limosilactobacillus albertensis* Lr3000^T

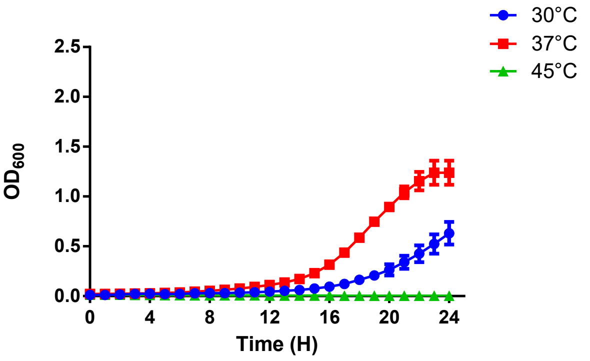

d. *Limosilactobacillus rudii* STM3_1^T

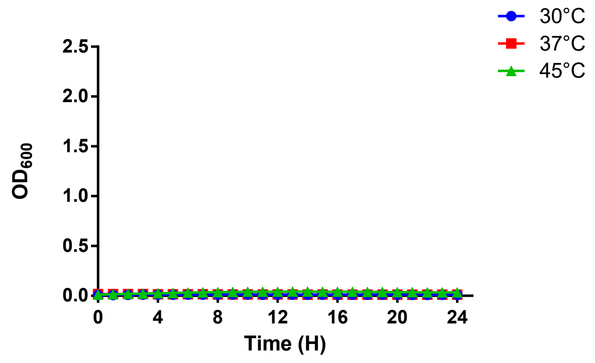

e. *Limosilactobacillus fastidiosus* WF-MO7-1^T

Fig S1. Growth curves of *Limosilactobacillus balticus* BG-AF3-A^T (**a**), *Limosilactobacillus agrestis* WF-MT5-A^T (**b**), *Limosilactobacillus albertensis* Lr3000^T (**c**), *Limosilactobacillus rudii* STM3_1^T (**d**), and *Limosilactobacillus fastidiosus* WF-MO7-1^T (**e**). Five putative type strains were incubated anaerobically in MRS broth (in the 96-well microplate; each well was overlaid with parafine to keep the oxygen out) at 15, 30, 37 and 45 °C, and OD_{600} was measured hourly using SpectraMax M3 Multi-Mode Microplate Readers (Molecular Devices, San Jose, CA, USA) for 24 hours. None of the strains grew at 15 °C.

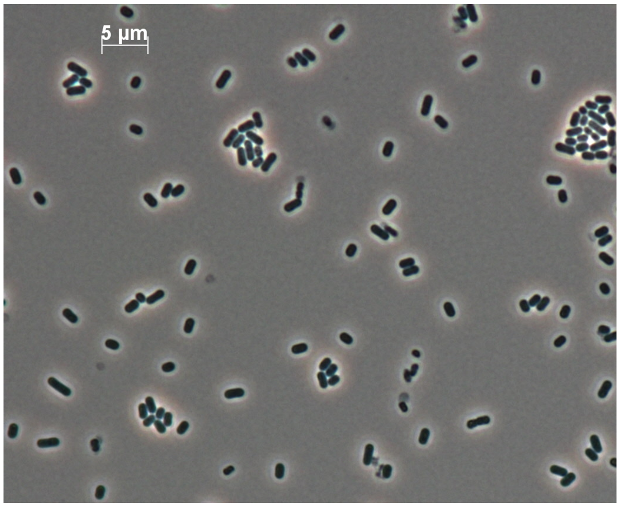

a. *Limosilactobacillus balticus* BG-AF3-A^T

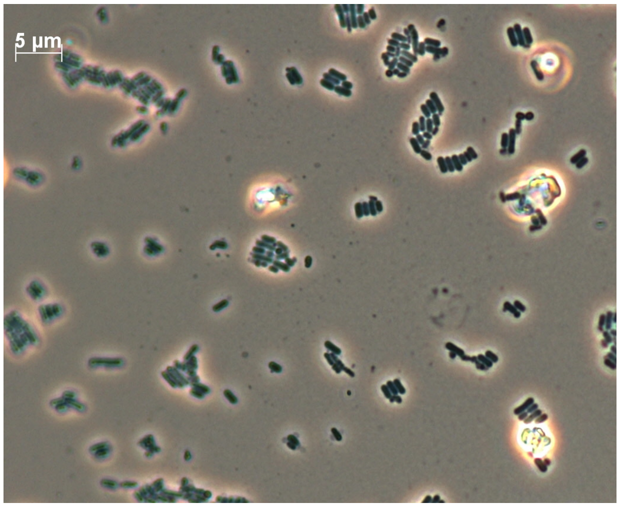

b. *Limosilactobacillus agrestis* WF-MT5-A^T

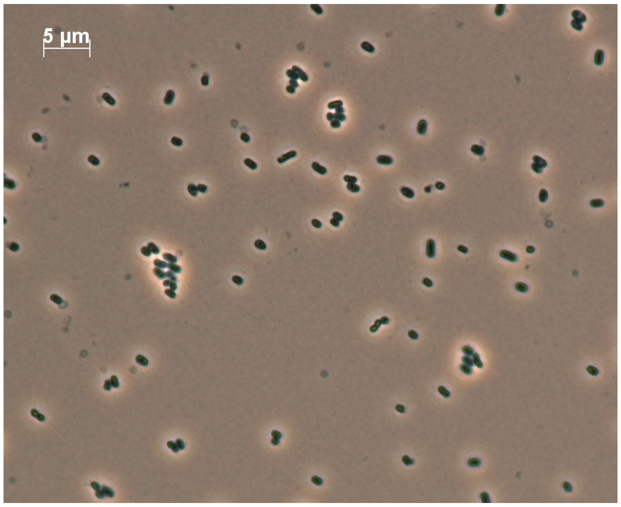

c. *Limosilactobacillus albertensis* Lr3000^T

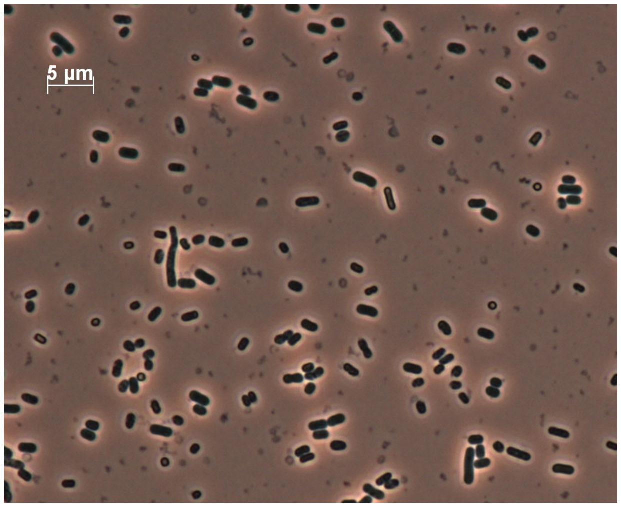

d. *Limosilactobacillus rudii* STM3_1^{T}

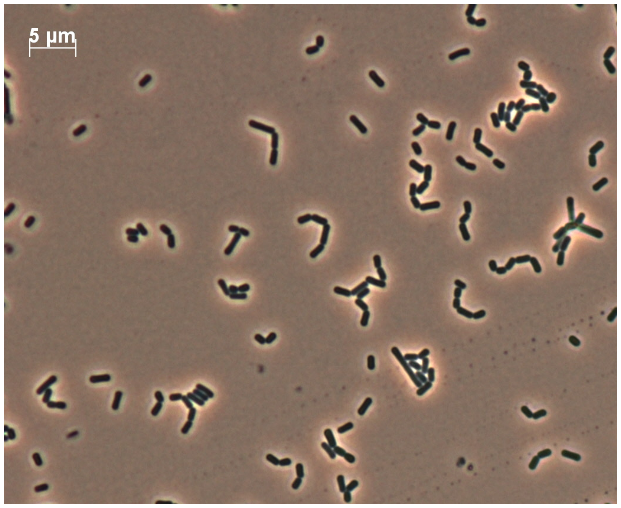

e. *Limosilactobacillus fastidiosus* WF-MO7-1^{T}

Fig S2. Cell morphology of *Limosilactobacillus balticus* BG-AF3-A^{T} (**a**), *Limosilactobacillus agrestis* WF-MT5-A^{T} (**b**), *Limosilactobacillus albertensis* Lr3000^{T} (**c**), *Limosilactobacillus rudii* STM3_1^{T} (**d**), and *Limosilactobacillus fastidiosus* WF-MO7-1^{T} (**e**). Cells of five putative type strains were grown on MRS agar plates for 24 - 48 hours at 28 °C under anaerobic conditions, and then were imaged with the ZEISS Scope-A1 microscope, which was carried out by the Identification Service of the DSMZ (Braunschweig, Germany).

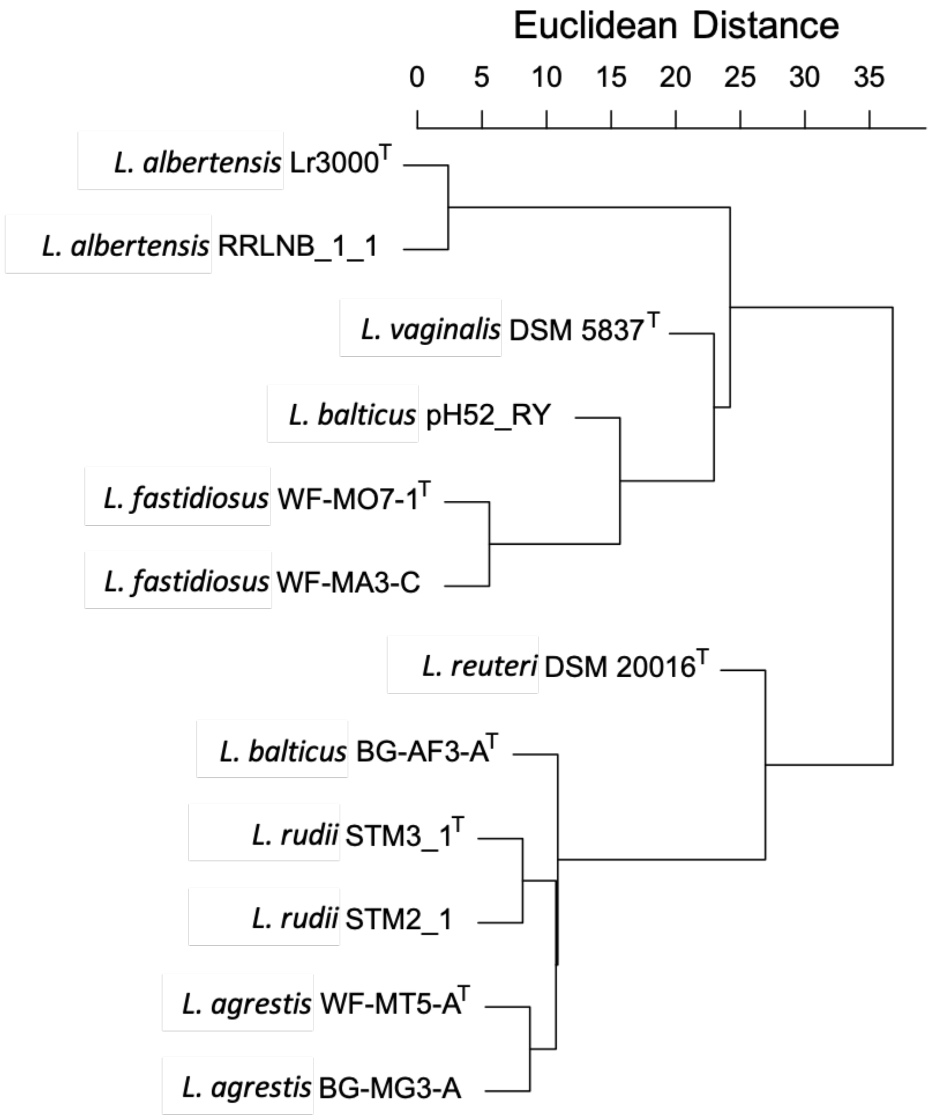

Fig S3. Cluster analysis of fatty acid compositions showing strains of *Limosilactobacillus* belonging the same novel species were generally more similar with each other, compared to strains from other novel species. Cluster analysis was conducted with Average clustering and Euclidean distance.

Table S1. *Limosilactobacillus reuteri* genomes used in this study

Strain	Origin	Lineage	Subspecies	Genome Accession Number
TD1	Rat	I	*Limosilactobacillus reuteri* ssp. *murium*	GenBank: GCA_000439275.1
I49	Mouse	I	*Limosilactobacillus reuteri* ssp. *murium*	JGI: 2718217763
LR0	Mouse	I	*Limosilactobacillus reuteri* ssp. *murium*	GenBank: GCA_002156605.1
LTH5448	Sourdough	I	*Limosilactobacillus reuteri* ssp. *murium*	JGI: 2571042361
lpuph1	Mouse	I	*Limosilactobacillus reuteri* ssp. *murium*	JGI: 2506381017
MM4-1a	Human	II	*Limosilactobacillus reuteri* ssp. *reuteri*	JGI: 2502171170
IRT	Human	II	*Limosilactobacillus reuteri* ssp. *reuteri*	JGI: 2636415954
JCM 1112	Human	II	*Limosilactobacillus reuteri* ssp. *reuteri*	GenBank: GCA_000010005.1
DSM 20016	Human	II	*Limosilactobacillus reuteri* ssp. *reuteri*	GenBank: GCA_000016825.1
MM2-3	Human	II	*Limosilactobacillus reuteri* ssp. *reuteri*	JGI: 2502171171
LR17	Horse	II	*Limosilactobacillus reuteri* ssp. *reuteri*	GenBank: GCA_003174915.1
LR10	Cow	II	*Limosilactobacillus reuteri* ssp. *reuteri*	GenBank: GCA_003175075.1
LR2	Goat	II	*Limosilactobacillus reuteri* ssp. *reuteri*	GenBank: GCA_003175015.1
LR8	Sheep	II	*Limosilactobacillus reuteri* ssp. *reuteri*	GenBank: GCA_003174875.1
LR19	Horse	II	*Limosilactobacillus reuteri* ssp. *reuteri*	GenBank: GCA_003175025.1
mlc3	Mouse	III	*Limosilactobacillus reuteri* ssp. *rodentium*	JGI: 2506381016
R2lc	Rat	III	*Limosilactobacillus reuteri* ssp. *rodentium*	GenBank: GCA_003703875.1
111b	Mouse	III	*Limosilactobacillus reuteri* ssp. *rodentium*	GenBank: GCA_002253965.1
TMW1.656	Sourdough	III	*Limosilactobacillus reuteri* ssp. *rodentium*	GenBank: GCA_000712565.2
100-23	Rat	III	*Limosilactobacillus reuteri* ssp. *rodentium*	GenBank: GCA_000168255.1
I5007	Pig	IV	*Limosilactobacillus reuteri* ssp. *suis*	GenBank: GCA_000410995.1
lp167-67	Pig	IV	*Limosilactobacillus reuteri* ssp. *suis*	JGI: 2599185361
ZLR003	Pig	IV	*Limosilactobacillus reuteri* ssp. *suis*	JGI: 2687453552
pg-3b	Pig	IV	*Limosilactobacillus reuteri* ssp. *suis*	JGI: 2599185334
ATCC 53608	Pig	IV	*Limosilactobacillus reuteri* ssp. *suis*	GenBank: GCA_000236455.2
3c6	Pig	V	*Limosilactobacillus reuteri* ssp. *porcinus*	JGI: 2599185333
20-2	Pig	V	*Limosilactobacillus reuteri* ssp. *porcinus*	JGI: 2599185332
SD2112	Human	VI	*Limosilactobacillus reuteri* ssp. *kinnaridis*	GenBank: GCA_000159455.2
CF48-3A	Human	VI	*Limosilactobacillus reuteri* ssp. *kinnaridis*	JGI: 2502171173
M27U15	Human	VI	*Limosilactobacillus reuteri* ssp. *kinnaridis*	JGI: 2687453659
JCM1081	Chicken	VI	*Limosilactobacillus reuteri* ssp. *kinnaridis*	JGI: 2684623011
CSF8	Chicken	VI	*Limosilactobacillus reuteri* ssp. *kinnaridis*	JGI: 2684623009
AP3	Pheasant	VI	*Limosilactobacillus reuteri* ssp. *kinnaridis*	GenBank: GCA_014145445.1
